# Restructuring of PM_2.5_ Composition and Source Contributions Across COVID-19 Social Distancing Periods: A Case Study at an Urban Monitoring Site in Seoul, South Korea

**DOI:** 10.3390/toxics14100886

**Published:** 2026-10-01

**Authors:** Shin-Young Park, Jaeheon Jung, Hyunsuk Choi, Jinyoung Yang, Yong-suk Choi, Cheol-Min Lee

**Affiliations:** 1 Department of Environmental & Chemical Engineering, Seokyeong University, Seoul 02713, Republic of Korea; tlsdud060900@skuniv.ac.kr; 2 Climate and Air Quality Team, Department of Atmospheric Environment Research, Research Institute of Public Health and Environment, Seoul 13818, Republic of Korea; jhjung1014@seoul.go.kr (J.J.); chs337@seoul.go.kr (H.C.); 53432@seoul.go.kr (J.Y.); hozer87@seoul.go.kr (Y.-s.C.)

**Keywords:** PM_2.5_, COVID-19, social distancing, positive matrix factorization (PMF), secondary inorganic aerosol, Seoul

## Abstract

This study examined whether changes in social activity associated with the Republic of Korea’s COVID-19 social distancing measures were accompanied by a simple reduction and recovery of PM_2.5_ or by source-specific temporal restructuring. Observations collected at an urban monitoring site in Gwangjin-gu, Seoul, from February 2018 to March 2024 were classified into pre-distancing (pre-D), social distancing (during-D), and post-distancing (post-D) periods. PM_2.5_ mass, chemical composition, secondary aerosol indicators, and PMF-derived source contributions were compared across periods, and generalized additive models were additionally applied to account for meteorological and seasonal variability. PM_2.5_ concentrations remained significantly lower during both the during-D and post-D periods than during the pre-D period after meteorological adjustment. Seven PMF sources were resolved. Secondary inorganic aerosols remained the dominant source and showed no significant period effect after adjustment. Metal-related industry decreased during the during-D period and partially rebounded during the post-D period, while Cr-rich industry and mixed combustion remained lower than during the pre-D period. Construction dust remained elevated during both the during-D and post-D periods, whereas paved road dust showed a significant increase during the post-D period after meteorological adjustment. These findings indicate source-specific temporal restructuring of PM_2.5_ at the study site rather than a uniform reduction-and-recovery pattern. The results highlight the importance of source-oriented management targeting secondary aerosol precursors, non-exhaust traffic emissions, construction-related fugitive dust, and industrial sources.

## 1. Introduction

Coronavirus disease 2019 (COVID-19), caused by severe acute respiratory syndrome coronavirus 2 (SARS-CoV-2), rapidly spread worldwide and was declared a global pandemic by the World Health Organization in March 2020 [1]. In response, governments implemented a range of public health measures, including restrictions on mobility, gatherings, commercial operations, and industrial activities, to reduce viral transmission. The implementation of these measures represented a major external intervention that abruptly altered the intensity of anthropogenic activities in urban environments, including road traffic, industrial operations, and commercial activities [2,3]. These restrictions on social activity are likely to affect not only the absolute concentrations of air pollutants but also the relative contributions of individual emission sources [4,5]. Consequently, numerous studies worldwide examined pandemic-related changes in air quality, with most reporting substantial reductions in traffic-related pollutants, particularly NO_2_, as well as decreases in PM_2.5_ and PM_10_ during periods of restricted social and economic activity [6,7]. Consistent with these global observations, studies conducted in South Korea reported decreases in PM_2.5_, PM_10_, NO_2_, and CO following the implementation of social distancing measures, particularly for pollutants associated with traffic and industrial activities [8,9].

The implementation of nationwide COVID-19 social distancing measures in South Korea represented a major exogenous intervention that altered the intensity and organization of urban activities, including human mobility, commercial activity, and industrial operations [9,10]. Unlike countries such as Italy, France, and India, which implemented strict nationwide lockdowns involving stay-at-home orders and extensive restrictions on mobility and non-essential activities [11,12,13], South Korea adopted a phased and adaptive social distancing strategy that repeatedly adjusted restrictions on gatherings, business operating hours, and daily mobility while allowing much of the urban system to remain operational [14,15]. This gradual modulation of social activity created a more complex transition than a simple shutdown-and-reopening sequence; therefore, the progressive resumption of social activities after the relaxation of distancing measures cannot be assumed to have resulted in an immediate return of the PM_2.5_ source contribution structure to its pre-distancing state. Rather, because mobility patterns, industrial operations, lifestyles, and energy-use structures may have recovered at different rates or remained altered after restrictions were lifted, the post-distancing period may have been characterized by a source-specific restructuring of the urban PM_2.5_ system rather than a simple restoration of pre-distancing conditions [16].

Accordingly, reductions in primary emissions associated with changes in the intensity of anthropogenic activities may not translate directly into proportional changes in PM_2.5_ mass concentration or chemical composition. As the abundance of primary emission-related components decreases, secondary components formed through atmospheric photochemical reactions and gas–particle partitioning processes may become relatively more important [17,18]. Secondary organic carbon (SOC), commonly estimated using the OC/EC method, and secondary inorganic aerosol (SIA), primarily comprising sulfate, nitrate, and ammonium, are widely used to evaluate the relative importance of secondary aerosol formation in PM_2.5_ [19,20]. Examining these secondary aerosol indicators across the pre-distancing, social distancing, and post-distancing periods can therefore help determine whether changes in social activity were accompanied by corresponding changes in atmospheric transformation processes. Thus, reductions in primary-source contributions may be accompanied by an increase in the relative importance of SOC and SIA, even when total PM_2.5_ concentrations decrease [21].

Previous studies have examined changes in PM_2.5_ chemical composition and source contributions before and during COVID-19 lockdowns or during the initial recovery phase after restrictions were relaxed [22,23,24]. However, most of these studies focused on short-term lockdown episodes or defined the post-restriction period using only several months or a single subsequent year, leaving uncertainty as to whether the chemical composition and source contribution structure of urban PM_2.5_ returned to their pre-distancing conditions over a longer recovery period or underwent persistent source-specific restructuring. A similar limitation applies to studies conducted in South Korea. Although previous research has documented changes in PM_2.5_ concentrations and chemical components during social distancing and the initial post-distancing period, and several studies have applied positive matrix factorization to identify major urban PM_2.5_ sources [25,26,27,28,29,30], few studies have compared the pre-distancing, social distancing, and extended post-distancing periods using long-term observations and a consistent source-apportionment framework. Whether individual source contributions returned to their pre-distancing levels or followed divergent trajectories after the lifting of social distancing measures remains insufficiently understood.

Therefore, this study aimed to evaluate temporal changes in the chemical composition and source contribution structure of PM_2.5_ associated with changes in social activity under South Korea’s COVID-19 social distancing framework, using long-term observations from an urban monitoring site in Gwangjin-gu, Seoul, as a case study. The study period was classified into pre-distancing, social distancing, and post-distancing periods according to the implementation and subsequent lifting of national social distancing measures in South Korea. PM_2.5_ mass concentration, chemical composition, secondary aerosol indicators, and PMF-derived source contributions were quantitatively compared across these periods to determine whether they returned to pre-distancing conditions after restrictions were lifted or followed source-specific trajectories indicative of restructuring in the PM_2.5_ source contribution structure at the study site.

## 2. Materials and Methods

### 2.1. Sampling Information

#### 2.1.1. Sampling Site

**Figure 1:**
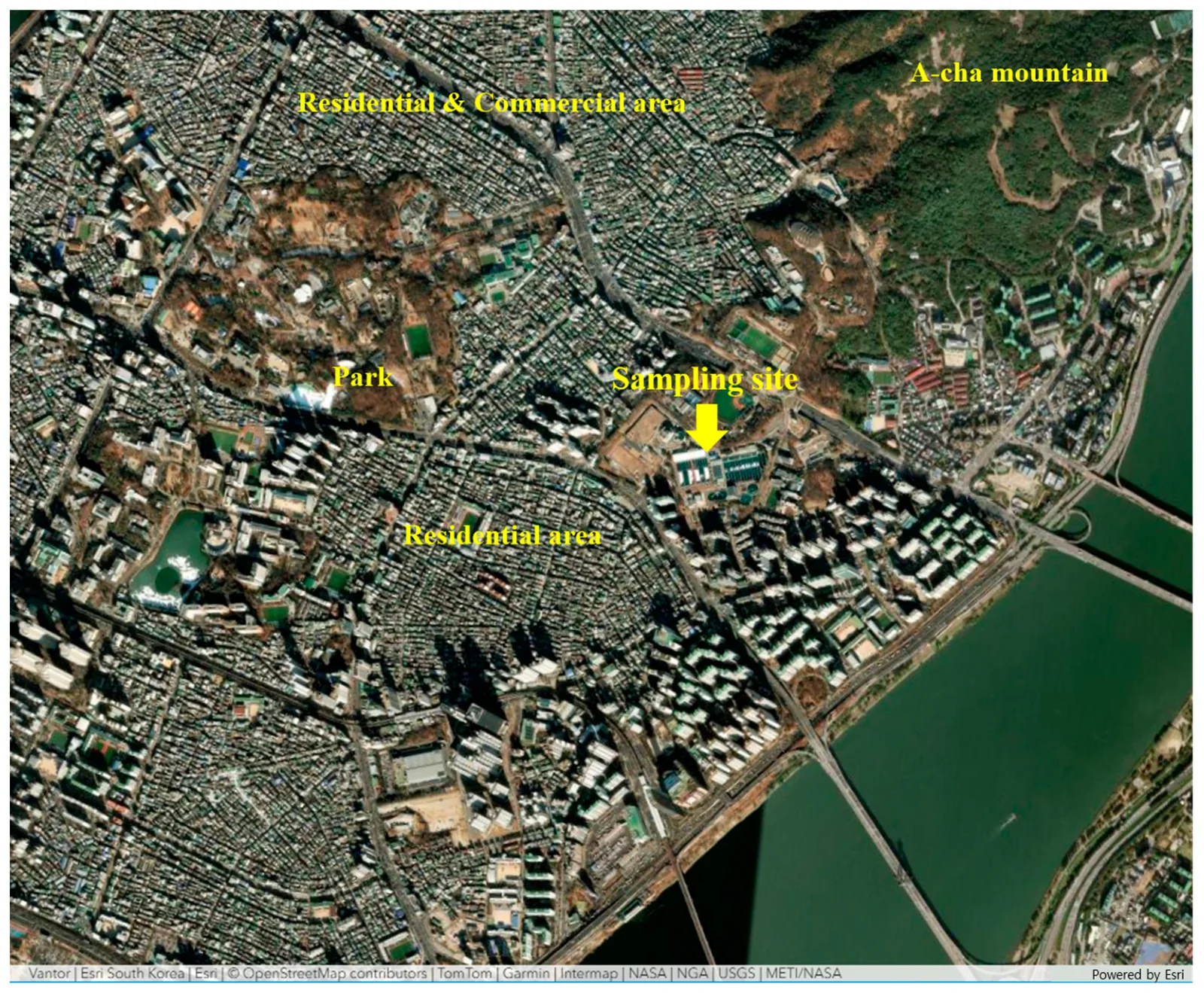
Location of the PM_2.5_ sampling site in Gwangjin-gu, Seoul, Republic of Korea. Background imagery was obtained from Esri World Imagery [33].

This study used chemical composition data for PM_2.5_ collected from an urban air quality monitoring station located in Gwangjin-gu, Seoul, Republic of Korea (37.5470° N, 127.0925° E) (Figure 1). The monitoring station is located in one of Korea’s representative urban areas, and intensive monitoring of PM_2.5_ has been conducted at this site because relatively high PM_2.5_ concentrations have been observed compared with other urban air quality monitoring stations in Seoul [31,32]. Residential areas, schools, and public facilities are distributed around the monitoring station, and subway stations and major roads are located within a 1 km radius. Therefore, the site is characterized by the combined influence of residential and commercial activities, as well as traffic-related emissions.

#### 2.1.2. Sampling and Chemical Analysis Method

PM_2.5_ samples were collected approximately 12 times per month from February 2018 to March 2024 using a low-volume air sampler, and a total of 870 samples were used for analysis. The target chemical components included eight ionic species (Cl^−^, NO_3_^−^, SO_4_^2−^, Na^+^, NH_4_^+^, K^+^, Ca^2+^, and Mg^2+^), two carbonaceous components (OC and EC), and 12 metallic elements (Be, Mg, Al, Cr, Mn, Fe, Ni, Cu, As, Cd, Pb, and Ca). Sample collection and chemical analysis were conducted in accordance with the operational standards of the national air quality monitoring network [32]. Detailed sampling conditions, component-specific analytical methods, and quality assurance/quality control (QA/QC) procedures are provided in the Supplementary Materials (Supplementary Notes S1 and S2, Tables S1 and S2).

### 2.2. Period Classification Based on COVID-19 Social Distancing Measures

In this study, PM_2.5_ chemical composition data were reclassified by period based on the implementation history of social distancing measures announced by the Korea Ministry of Health and Welfare [34]. Accordingly, the study period was divided into the pre-distancing (pre-D), social distancing (during-D), and post-distancing (post-D) periods. The social distancing period was defined as the interval from the introduction of the “distancing in daily life” system through the period during which national social distancing measures remained in effect. The period beginning on 18 April 2022, when major mandatory social distancing measures were lifted as part of the transition toward routine disease management, was classified as the post-distancing period. Detailed definitions and dates for each period are provided in Table S3.

During the during-D period, the level and intensity of social-distancing measures were repeatedly adjusted according to epidemic conditions. Major measures included restrictions on the size of private gatherings, operating-hour and capacity limits for restaurants, cafés, indoor sports facilities, entertainment venues, and other multi-use facilities, as well as restrictions on large events and religious gatherings [35]. In the Seoul metropolitan area, stricter measures were intermittently implemented, including reduced gathering sizes and shortened business operating hours. These restrictions were progressively relaxed, and most social-distancing measures were lifted on 18 April 2022 [34].

### 2.3. Assessment of Secondary Aerosol Contributions and Source Apportionment

#### 2.3.1. Assessment of Secondary Aerosol Contributions

PMF is useful for identifying major PM_2.5_ sources and estimating their contributions; however, because it does not explicitly simulate atmospheric chemical reactions or transformation processes, it has limitations in independently quantifying the formation characteristics of secondary aerosols [36,37]. Therefore, in this study, secondary organic carbon formation, expressed as secondary OC (OC_sec_), the sulfur oxidation ratio (SOR), and the nitrogen oxidation ratio (NOR) were calculated to evaluate the secondary formation characteristics of PM_2.5_ [38,39,40,41]. The calculation equations are shown in Equations (1)–(3).

OC_sec_ = OC_Tot_ − EC × (OC/EC)_pri_ (1)

SOR = nSO_4_^2−^/(nSO_4_^2−^ + nSO_2_) (2)

NOR = nNO_3_^−^/(nNO_3_^−^ + nNO_2_) (3)

where OC_Tot_ and EC represent the concentrations of total OC and EC, respectively, in μg/m^3^, and (OC/EC)_pri_ represents the primary OC/EC reference ratio used in the EC-tracer method. To account for seasonal variability in primary carbonaceous aerosol characteristics, the season-specific 5th percentile of the observed OC/EC ratio was used as (OC/EC)_pri_ (Table S4). Negative OC_sec_ estimates were set to zero. The calculated OC_sec_ was interpreted as a semi-quantitative estimate of OC attributable to secondary formation. The terms nSO_4_^2−^, nSO_2_, nNO_3_^−^, and nNO_2_ represent the molar concentrations of SO_4_^2−^, SO_2_, NO_3_^−^, and NO_2_, respectively, in mol/m^3^. SOR and NOR were used as indicators of the relative oxidative conversion of SO_2_ to sulfate and NO_2_ to nitrate, respectively, with higher values indicating greater relative conversion of gaseous precursors to secondary particulate species. Because OC_sec_ estimates obtained using the EC-tracer method are sensitive to the assumed primary OC/EC reference ratio, a sensitivity analysis was additionally performed using the season-specific minimum and 10th percentile OC/EC ratios as alternative reference scenarios. OC_sec_ was recalculated under each scenario, and the resulting period-specific estimates and EC–OC_sec_relationships were compared with those obtained using the 5th-percentile reference values.

#### 2.3.2. Source Apportionment

PMF is a receptor model that simultaneously estimates source composition profiles and source contributions using observed concentrations and associated uncertainties without requiring prior source profile information [42]. It has been widely applied to quantitatively identify major sources of atmospheric PM_2.5_ [43,44,45]. In this study, PMF version 5.0, provided by the U.S. Environmental Protection Agency, was applied to classify PM_2.5_ sources and estimate their contributions.

The PMF input data and uncertainty data were constructed based on PM_2.5_ mass concentrations and chemical component concentrations. Concentrations below the method detection limit (MDL) were replaced with MDL/2, and their corresponding uncertainties were assigned as 5/6 × MDL following the established PMF treatment for below-detection-limit observation [46,47,48]. However, species with detection rates below 50% were excluded from the analysis and were not used for PMF modeling [46,47,48]. For values above the MDL, uncertainty was calculated using the error fraction (EF) and concentration (Equation (4)). In this study, the EF was set to 10% [46,49,50], and an additional modeling uncertainty of 20% was applied.

(4) $$\mathrm{Unc}=\sqrt{({\mathrm{EF}\times C)}^{2}+({0.5\times \mathrm{MDL})}^{2}}$$

This uncertainty treatment reduced the statistical weight of observations with greater uncertainty, particularly low-concentration measurements near or below the MDL, thereby limiting their disproportionate influence on the PMF solution [46,47]. Accordingly, greater assigned uncertainty decreases the relative influence of small concentration variations on the PMF solution [47].

Before PMF analysis, the signal-to-noise ratio (S/N ratio) of each chemical species was evaluated for data quality control. Species with S/N < 0.5 were classified as “Bad” and excluded from the analysis. Species with 0.5 ≤ S/N < 1.0 were classified as “Weak”, whereas those with S/N ≥ 1.0 were classified as “Strong”. Species classified as Weak were included in the model calculation, but their uncertainties were adjusted to prevent their influence from being overrepresented in the model results.

Before determining the optimal number of PMF factors, principal component analysis (PCA) was performed to estimate the potential number of sources. Varimax rotation was applied to PCA to improve the interpretability of factor structures and to more clearly distinguish the factor loadings of each chemical species [51]. The potential number of factors was initially selected based on a cumulative explained variance of at least 70% and eigenvalues greater than 1.0 [52,53]. Subsequently, PMF models were sequentially run using factor numbers within ±2 of the number of factors derived from PCA. The final number of factors was determined by comprehensively considering the Q values, distribution of scaled residuals, agreement between observed and predicted values, and the physical and chemical interpretability of the resolved source profiles. The stability and rotational uncertainty of the selected seven-factor pre-D PMF solution were further evaluated using the bootstrap (BS) and displacement (DISP) error-estimation methods implemented in EPA PMF 5.0. Bootstrap analysis was conducted using 100 resamples with a minimum Pearson correlation coefficient of 0.6 for factor mapping. DISP analysis was performed at dQmax values of 4, 8, 15, and 25 to evaluate potential factor swaps and rotational ambiguity. The resulting bootstrap mapping rates and DISP diagnostics were used to assess the reproducibility and stability of the resolved source factors.

PMF analysis was conducted in two steps to evaluate temporal changes in source contributions across the pre-D, during-D, and post-D periods. First, PMF was applied to the pre-D dataset to identify the baseline source structure and determine the optimal number of factors and source interpretation criteria. Second, the integrated dataset covering the entire monitoring period from February 2018 to March 2024 was analyzed using the same number of factors as identified in the pre-D analysis to facilitate comparison of source contributions across the three study periods. To assign source identities in the integrated PMF solution, the integrated factors were matched to the baseline pre-D factors by jointly considering Pearson correlations between factor contribution time series over the overlapping pre-D samples, similarities in major chemical marker profiles, and source interpretability. To further evaluate the correspondence between the two PMF solutions, source contribution fractions from the integrated PMF were recalculated using only the overlapping pre-D samples and directly compared with those obtained from the pre-D-only PMF solution. This approach enabled temporal changes in source contributions to be evaluated within a common integrated PMF solution while also assessing the consistency and uncertainty of source correspondence between the two PMF analyses.

### 2.4. Statistical Analysis

#### 2.4.1. General Statistical Analysis

General statistical analyses were performed using SPSS (version 23.0; IBM Corp., Armonk, NY, USA). Differences in PM_2.5_ mass concentrations and chemical component concentrations among the pre-D, during-D, and post-D periods were evaluated using the Kruskal–Wallis test, followed by Dunn’s post hoc test. Correlations among variables were evaluated using Pearson correlation analysis. The statistical significance level was set at α = 0.05.

#### 2.4.2. Meteorological Adjustment Using Generalized Additive Models

To assess the potential influence of meteorological variability on the observed differences among the pre-D, during-D, and post-D periods, meteorological conditions during the study period were first characterized and subsequently incorporated into generalized additive models (GAMs) as covariates. Daily meteorological data were obtained from the Automated Synoptic Observing System (ASOS) operated by the Korea Meteorological Administration [54]. The meteorological variables included daily mean temperature (°C), mean relative humidity (%), mean wind speed (m/s), daily precipitation (mm), and daily total solar radiation. Meteorological data were matched to each PM_2.5_ sample based on the corresponding calendar date. Blank precipitation records indicating no measurable precipitation were assigned a value of 0 mm, whereas missing observations for the other meteorological variables were retained as missing. Period-specific distributions of the meteorological variables were summarized to characterize meteorological variability across the three study periods, and differences among periods were evaluated using the Kruskal–Wallis test.

GAMs were then applied to evaluate whether the observed differences among the study periods remained after accounting for meteorological variability. Study period was included as a categorical explanatory variable, with the pre-D period used as the reference category. Daily mean temperature, relative humidity, wind speed, and solar radiation were incorporated as meteorological covariates using smooth functions to allow for potentially nonlinear associations. Because precipitation was highly right-skewed with a large proportion of zero values, it was transformed as log(1 + precipitation) and included as a linear covariate. Seasonal variability was additionally controlled using a cyclic smooth function of day of year (DOY). The general model structure was expressed as

g{E(*Y_i_*)} = *β*_0_ + *β*_1_ I(During − *D_i_*) + *β*_2_ I(Post − *D_i_*) + *s*_1_(*T_i_*) + *s*_2_(*RH_i_*) + *s*_3_(*WS_i_*) + *β*_3_log(1 + *P_i_*) + *s*_4_(*SR_i_*) + *s_cyc_*(*DOY_i_*) (5)

where *Y_i_* represents the outcome for sampling event *i*; *g*(⋅) denotes the link function; *I*(During − D) and *I*(Post − D) are indicator variables for the study period; and *T*, *RH*, *WS*, *P*, and *SR* represent daily mean temperature, relative humidity, wind speed, precipitation, and daily total solar radiation, respectively. Temperature, relative humidity, wind speed, and solar radiation were modeled using smooth functions to allow for potentially nonlinear associations. Because precipitation showed a highly right-skewed distribution with a large proportion of zero values, it was transformed as log(1 + *P*) and included as a linear covariate. *s*(⋅) denotes a smooth function, and *s*_cyc_(*DOY*) represents a cyclic smooth function of day of year used to account for recurring seasonal variation.

Separate GAMs were fitted for PM_2.5_ mass concentration, SOR, NOR, and each of the seven PMF-derived source contribution scores. PM_2.5_ was log-transformed and modeled using a Gaussian error distribution with an identity link. SOR and NOR, which were continuous variables bounded between 0 and 1 with no observations at the boundaries, were modeled using beta regression GAMs with a logit link. The seven PMF-derived source contribution scores were modeled using Gaussian GAMs with an identity link because the normalized factor scores included negative values. The coefficients for the during-D and post-D indicator variables represented meteorology-adjusted differences relative to the pre-D period. The post-D versus during-D difference was additionally evaluated using the contrast *β*_2_ − *β*_1_. Models were fitted using complete observations for the corresponding outcome and meteorological covariates. Because residual diagnostics indicated comparatively heavy-tailed distributions for the SIA and construction-dust models, additional scaled-t robust GAMs were fitted as sensitivity analyses to evaluate the robustness of the period-effect estimates. GAM analyses were performed in R (version 4.3.3) using the *mgcv* package (version 1.9-1).

## 3. Results and Discussion

### 3.1. Temporal Variations in PM_2.5_ and Chemical Composition Across Social Distancing Periods

#### 3.1.1. Temporal Variations in PM_2.5_

As shown in Table 1, the mean PM_2.5_ concentration was highest during the pre-D period (25.54 μg/m^3^), followed by the during-D (20.91 μg/m^3^) and post-D periods (19.28 μg/m^3^). The Kruskal–Wallis test revealed a significant difference among the three periods (*p* < 0.05). Subsequent Dunn’s post hoc test indicated that PM_2.5_ concentrations during both the during-D and post-D periods were significantly lower than those during the pre-D period, whereas no significant difference was observed between the during-D and post-D periods (*p* > 0.05). These findings suggest that PM_2.5_ concentrations did not immediately return to pre-distancing levels after the resumption of social activities.

#### 3.1.2. Temporal Changes in the Chemical Composition of PM_2.5_

As shown in Table 2, most chemical components of PM_2.5_ showed the highest concentrations during the pre-D period and generally decreased during the during-D and post-D periods. Among the ionic species, SO_4_^2−^, NH_4_^+^, and Cl^−^ showed significantly lower concentrations during the during-D and post-D periods than during the pre-D period (*p* < 0.05). K^+^ also exhibited a decreasing trend with statistically significant differences among the three periods (*p* < 0.05). In contrast, NO_3_^−^ showed no significant difference among the three periods (*p* > 0.05).

For carbonaceous components, both OC and EC showed the highest concentrations during the pre-D period and significantly lower levels during the during-D and post-D periods (*p* < 0.05). In particular, EC decreased from 0.95 μg/m^3^ during the pre-D period to 0.47 μg/m^3^ and 0.48 μg/m^3^ during the during-D and post-D periods, respectively (*p* < 0.05). No significant difference was observed between the during-D and post-D periods (*p* > 0.05). These results suggest that the influence of primary emissions was reduced after the onset of the during-D period and that this decreasing tendency persisted even after the end of social distancing measures.

For metallic elements, Al, Fe, Cu, Pb, Cd, and Cr showed significantly lower concentrations during the during-D and post-D periods than during the pre-D period (*p* < 0.05), whereas Mn showed no significant difference among the three periods (*p* > 0.05). In contrast, As exhibited a distinct temporal pattern, with the highest concentration observed during the post-D period (5.26 ng/m^3^; *p* < 0.05). As has been associated with non-ferrous metal smelting and other high-temperature industrial processes [55,56]. However, because facility-specific As emission data were not available for the monitoring site and surrounding area, the source responsible for the post-D period increase could not be directly identified. Therefore, the elevated As concentration during the post-D period is interpreted here as a distinct temporal pattern rather than as evidence of increased emissions from a specific industrial or combustion source.

In contrast, Ca^2+^ and Mg^2+^ tended to increase during the during-D and post-D periods compared to the pre-D period. Ca^2+^ and Mg^2+^ have been reported as major ionic species associated with soil mineral particles, road and surface resuspension, construction-related dust, and sea salt components [57,58,59]. Therefore, unlike the decrease in general combustion or industrial emissions, the increases in these species suggest that the influences of surface disturbance, construction and civil engineering activities, resuspension conditions, meteorological factors, and externally transported dust may have become relatively more pronounced after the during-D period [60,61].

Overall, the chemical composition of PM_2.5_ differed markedly across the pre-D, during-D, and post-D periods. Concentrations of SO_4_^2−^, NH_4_^+^, Cl^−^, K^+^, OC, EC, and several metallic elements were lower during during-D and post-D than during pre-D, indicating a reduced influence of combustion- and industry-related sources. In contrast, NO_3_^−^ showed no significant temporal change, indicating the relative persistence of secondary nitrate formation. Other components exhibited distinct temporal responses: As increased during post-D, whereas Ca^2+^ and Mg^2+^ were higher during during-D and post-D than during pre-D. Collectively, the results indicate that changes in social activity associated with the implementation and lifting of social distancing measures were accompanied not only by changes in PM_2.5_ mass concentration but also by a component-specific restructuring of its chemical composition, reflecting differences in source characteristics, atmospheric formation processes, and transport pathways.

### 3.2. Secondary Aerosol-Related Characteristics of PM_2.5_

#### 3.2.1. SOC Characteristics of PM_2.5_

**Figure 2:**
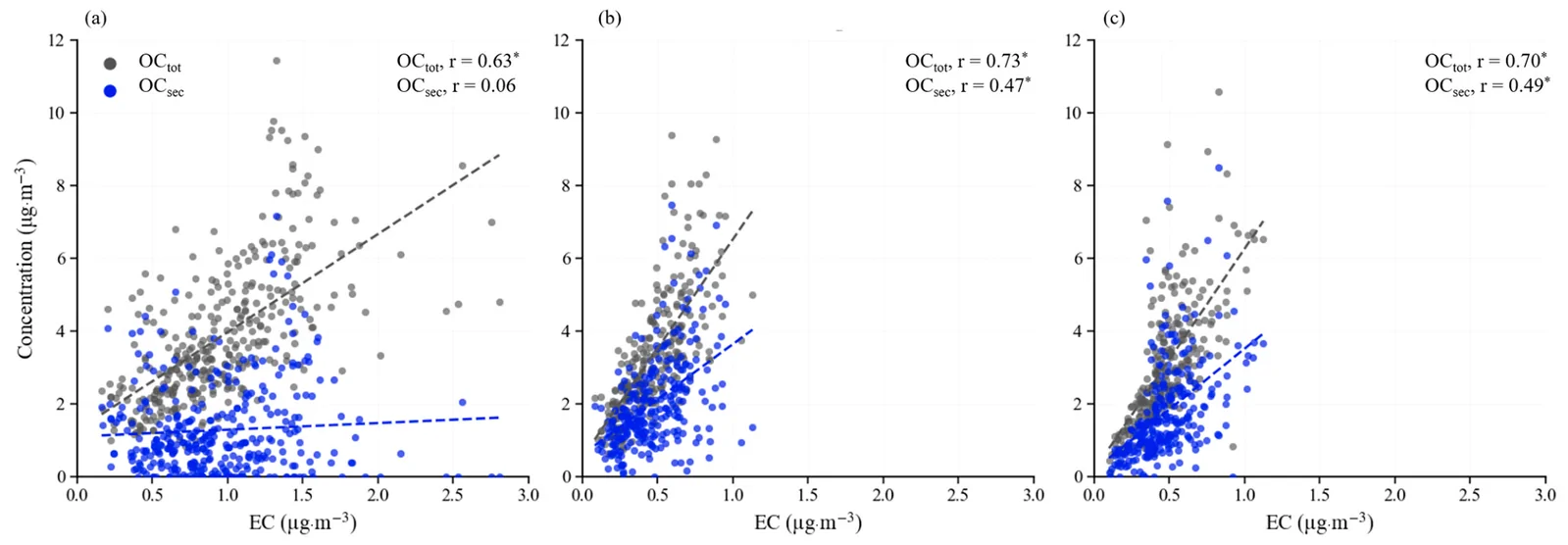
Scatter plots of EC versus OC_tot_ and OC_sec_ during pre-D (**a**), during-D (**b**), and post-D (**c**) periods. * *p*-value < 0.05.

The distribution characteristics of OC_Tot_, OC_sec_, and EC were compared across the periods (Figure 2). OC_Tot_ showed significant positive correlations with EC in all three periods (r = 0.63, 0.73, and 0.70, respectively), suggesting that primary combustion-derived carbonaceous components were consistently reflected in OC_Tot_ throughout the study period. Meanwhile, the slope between EC and OC_Tot_ increased from 2.70 in the pre-D period to 5.99 and 6.09 in the during-D and post-D periods, respectively (Table S5). This result indicates that the relative proportion of OC_Tot_ to EC increased after the during-D period, suggesting changes in the emission characteristics or compositional structure of carbonaceous components.

According to a previous study [30], the decrease in EC and increase in the OC/EC ratio during the post-D period were interpreted as reflecting not only a simple reduction in primary activities but also changes in emission characteristics and a relative increase in secondary organic aerosol formation. Therefore, the increased slope of OC_Tot_ relative to EC after the during-D period observed in this study also supports the possibility that, under reduced primary combustion-related emissions, the contribution of secondary OC or relatively oxidized organic carbonaceous components to residual OC increased.

For OC_sec_, no significant correlation with EC was observed during the pre-D period (*p* > 0.05), whereas significant positive correlations were found during the during-D and post-D periods, with correlation coefficients of 0.47 and 0.49, respectively (*p* < 0.05). The slope also increased markedly from 0.19 in the pre-D period to 3.04 and 3.33 in the during-D and post-D periods, respectively. These results suggest that, before during-D, OC_sec_ precursors originating from diverse sources may have contributed in a complex manner, resulting in no clear covariation with EC. In contrast, after the onset of during-D, changes in traffic- and combustion-related emissions may have altered the emission characteristics of atmospheric organic carbon, leading OC_sec_ to exhibit closer variability with EC. This indicates that OC_sec_ formation during the during-D and post-D periods may have been more closely associated with residual combustion-related emissions or their related precursors.

A sensitivity analysis was conducted to evaluate the uncertainty associated with the selection of the primary OC/EC reference ratio. Using the season-specific minimum and 10th-percentile OC/EC ratios as alternative reference scenarios, the absolute OC_sec_ estimates varied substantially (Table S6). Relative to the primary 5th-percentile method, mean OC_sec_ increased by 107.6%, 36.5%, and 41.1% under the minimum-ratio scenario for the pre-D, during-D, and post-D periods, respectively, whereas the corresponding estimates decreased by 21.2%, 8.6%, and 9.7% under the 10th-percentile scenario. Nevertheless, the during-D period consistently showed the highest mean OC_sec_ across all three reference scenarios. The positive relationships between EC and OC*_sec_* during the during-D and post-D periods also remained statistically significant under all sensitivity scenarios (Table S7). In contrast, the EC–OC_sec_ relationship during the pre-D period was sensitive to the selected reference ratio, becoming significant only when the seasonal minimum OC/EC ratio was used. These results indicate that the absolute magnitude of OC_sec_, particularly during the pre-D period, should be interpreted with caution.

#### 3.2.2. SIA Characteristics of PM_2.5_

**Figure 3:**
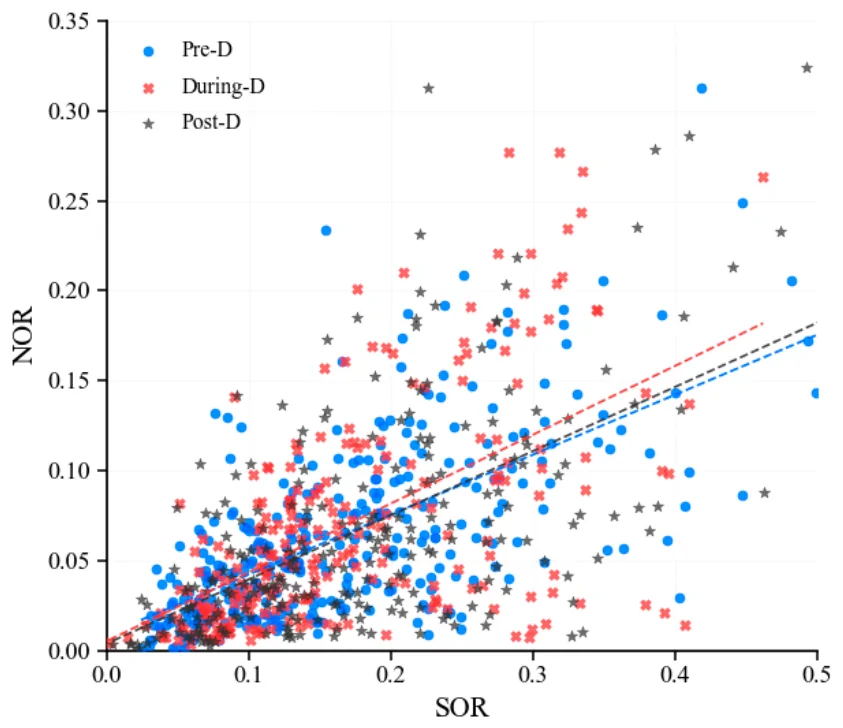
Scatter plots of SOR and NOR during pre-D, During-D, and post-D. *p* < 0.05. Dashed lines indicate the fitted regression lines for each period (blue: pre-D; red: during-D; gray: post-D). *p* < 0.05.

The distribution characteristics of SOR and NOR were compared across the periods (Figure 3). SOR values were 0.16 ± 0.10, 0.17 ± 0.09, and 0.18 ± 0.10 during the pre-D, during-D, and post-D periods, respectively, showing a slight increase after during-D; however, the overall differences among the periods were not substantial. NOR values were 0.06 ± 0.05, 0.07 ± 0.06, and 0.07 ± 0.06, respectively, indicating a slight increase during during-D and a similar level during post-D. As shown in Table S8, SO_2_ and SO_4_^2−^ decreased concurrently across the study periods, whereas NO_2_ decreased more markedly than NO_3_^−^, resulting in only minor variations in SOR and NOR. The persistence of NO_3_^−^, together with the relatively stable oxidation ratios, may also partly explain why the SIA contribution did not differ significantly among the three periods despite the lower total PM_2.5_ concentrations during the during-D and post-D periods. Overall, SOR values were higher than NOR values, suggesting that the secondary oxidation conversion of SO_2_ to SO_4_^2−^ was relatively more pronounced than the conversion of NO_2_ to NO_3_^−^ during the study period.

Significant positive correlations between SOR and NOR were observed in all three periods (pre-D: r = 0.65; during-D: r = 0.58; post-D: r = 0.59; all *p*-values < 0.05), indicating that the secondary formation of SO_4_^2−^ and NO_3_^−^ tended to increase together under similar atmospheric oxidation conditions. These unadjusted comparisons suggested relatively similar secondary inorganic aerosol transformation across periods; however, the meteorology-adjusted analysis showed significantly lower SOR during the during-D and post-D periods, whereas NOR remained unchanged.

### 3.3. Identification of PM_2.5_ Sources and Temporal Changes in Source Contributions

#### 3.3.1. Identification of PM_2.5_ Sources During Pre-Distancing Period

**Figure 4:**
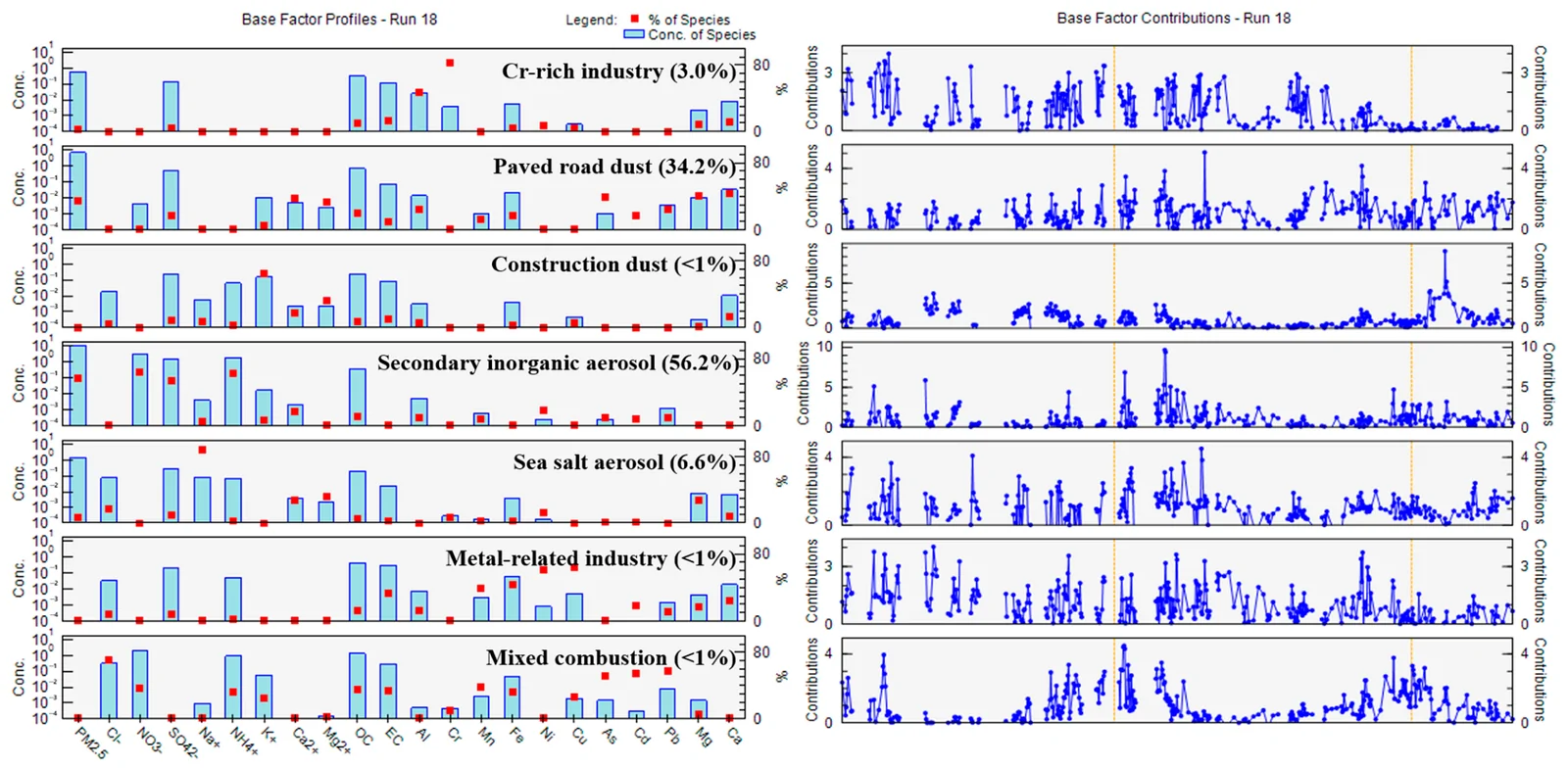
Source profiles and time-series contributions of the PM_2.5_ factors resolved by PMF using the pre-D dataset. The yellow dashed vertical lines indicate the boundaries between calendar years.

PMF analysis was conducted in two steps to evaluate temporal changes in source contributions relative to the pre-D baseline. First, PMF was applied to the pre-D dataset to identify the baseline source structure under pre-D emission conditions. Based on the PCA results and PMF diagnostic parameters (Tables S9 and S10), seven sources were selected as the optimal solution (Figure 4, Table S11).

The first factor was characterized by a high relative contribution of Cr, along with carbonaceous components and several metallic elements, including Al, Fe, Mg, and Ca. Previous source-apportionment studies have identified Cr together with other metals such as Fe, Ni, Mn, Cu, and Pb in factors associated with steelmaking and metallurgical activities [62]. Therefore, this factor was interpreted as a Cr-rich industrial factor.

The second factor accounted for 34.2% of total PM_2.5_ and was characterized by PM_2.5_, OC, EC, Fe, Mg, Ca, Pb, and other crustal and traffic-related elements. This profile was consistent with paved road dust, which is represented in the U.S. EPA SPECIATE database as a road dust profile generated by grab/vacuum sampling and resuspension methods (U.S. EPA SPECIATE v5.4, Profile code 3661). Therefore, this factor was interpreted as paved road dust, reflecting the influence of resuspended road surface particles and traffic-related non-exhaust emissions.

The third factor was characterized by mineral-related components, including Na^+^, K^+^, Ca^2+^, Mg^2+^, and Ca, and showed episodic contributions concentrated during specific periods. These features are consistent with the characteristics of dust generated from construction and local surface disturbance. Therefore, this factor was interpreted as construction dust (U.S. EPA SPECIATE v5.4, Profile code 2720410). Urban redevelopment and construction activities near the monitoring site were considered likely contributors to this source.

The fourth factor was dominated by secondary inorganic aerosol components, particularly NO_3_^−^, SO_4_^2−^, and NH_4_^+^, and showed the largest contribution to total PM_2.5_, accounting for 56.2%. Because this factor included both nitrate-, ammonium-, and sulfate-related ions, it was interpreted as secondary inorganic aerosol rather than as secondary nitrate alone [63,64,65]. The high contribution of this factor indicates that PM_2.5_ at the monitoring site was strongly influenced by secondary aerosol formation during the study period.

The fifth factor was characterized by relatively high contributions of Na+ and Cl-, which are representative tracers of sea-salt aerosol. This factor was therefore interpreted as sea-salt aerosol based on its similarity to the corresponding profile in the U.S. EPA SPECIATE database (v5.4, Profile code 44310110). Backward-trajectory analysis of the five pre-D sampling days with the highest sea-salt factor contribution scores showed that air masses generally approached the monitoring site from the western sector, spanning northwestern to southwestern pathways (Supplementary Notes S3). Four of the five trajectories passed over the Yellow Sea or adjacent marine areas west of the Korean Peninsula before reaching Seoul, whereas one case showed a local/regional recirculation pattern (Table S12; Figure S1). These trajectory patterns support the potential influence of regional marine aerosol transport on the resolved sea-salt factor.

The sixth factor was characterized by relatively high contributions of metallic elements, including Fe, Ni, Cu, As, Cd, and Pb. Although this factor contributed less than 1% to total PM_2.5_ mass, it explained meaningful variability in trace metal components. Therefore, this factor was interpreted as a metal-related industrial source.

The seventh factor was characterized by Cl^−^, NO_3_^−^, NH_4_^+^, K^+^, OC, EC, and several trace metals, including Cu, As, Cd, and Pb. The co-occurrence of combustion-related species, such as Cl^−^, K^+^, OC, and EC, may indicate the influence of biomass burning, waste incineration, or other urban combustion activities (U.S. EPA SPECIATE v5.4, Profile codes 1710510 and 3766AE6). The presence of trace metals further suggests possible contributions from combustion-related industrial processes. Because this factor could not be assigned to a single source, it was interpreted as a mixed combustion source. Although its contribution to total PM_2.5_ mass was less than 1%, this factor was retained because it explained part of the covariance structure of combustion-related and trace metal species.

Overall, secondary inorganic aerosols were the dominant contributor to PM_2.5_, accounting for 56.2% of total mass, followed by paved road dust at 34.2%. These results indicate that PM_2.5_ at the monitoring site was mainly influenced by secondary aerosol formation and resuspended road dust. Sea-salt aerosol and the Cr-rich industry contributed 6.6% and 3.0%, respectively, whereas construction dust, metal-related industry, and mixed combustion each contributed less than 1%. Error-estimation analyses further supported the reproducibility of the resolved seven-factor structure (Table S13). Bootstrap mapping rates were 100% for the Cr-rich industrial factor, 99% for the metal-related industrial factor, and 97% for mixed combustion, despite their relatively small contributions to total PM_2.5_ mass. Paved road dust, sea-salt aerosol, and SIA also showed mapping rates of 89%, 100%, and 85%, respectively. In addition, the DISP analysis showed a largest decrease in Q of 0.000, with no factor swaps at dQmax values of 4, 8, 15, or 25, indicating limited rotational ambiguity. In contrast, construction dust showed a lower bootstrap mapping rate of 48%, suggesting comparatively greater uncertainty in this factor and warranting more cautious interpretation. The backward-trajectory results further indicated that the sea-salt factor was frequently associated with air masses approaching Seoul through the western marine sector.

#### 3.3.2. Integrated Source Apportionment and Comparison of Source Contributions Across Periods

Based on the seven-factor solution identified in the pre-D analysis, the entire dataset, including the pre-D, during-D, and post-D samples, was analyzed using the same number of factors to enable consistent comparison across the social distancing periods (Tables S14 and S15). Integrated PMF factors were matched with the initial pre-D PMF factors by considering source–contribution correlations, major chemical marker profiles, and source interpretability, with the correlation matrix presented in Figure S2. To further evaluate the consistency between the two PMF solutions, source contribution fractions from the integrated PMF were recalculated using only the overlapping pre-D samples and directly compared with those from the pre-D-only PMF solution (Table S16).

As shown in Figures S3 and S4, source contributions exhibited source-specific temporal patterns. Secondary inorganic aerosol was the dominant contributor, accounting for 42.5% of total PM_2.5_, indicating the persistent importance of secondary aerosol formation throughout the study period. Metal-related industry, sea-salt aerosol, and paved road dust contributed 28.0%, 11.0%, and 11.1%, respectively, with paved road dust showing episodic peaks during during-D and post-D. Construction dust contributed 7.4% and showed intermittent high-contribution episodes after the onset of social distancing, suggesting the influence of local construction or surface disturbance activities. In contrast, Cr-rich industry and mixed combustion each contributed less than 1% and were interpreted as minor but chemically meaningful factors associated with trace metal and combustion-related species.

**Figure 5:**
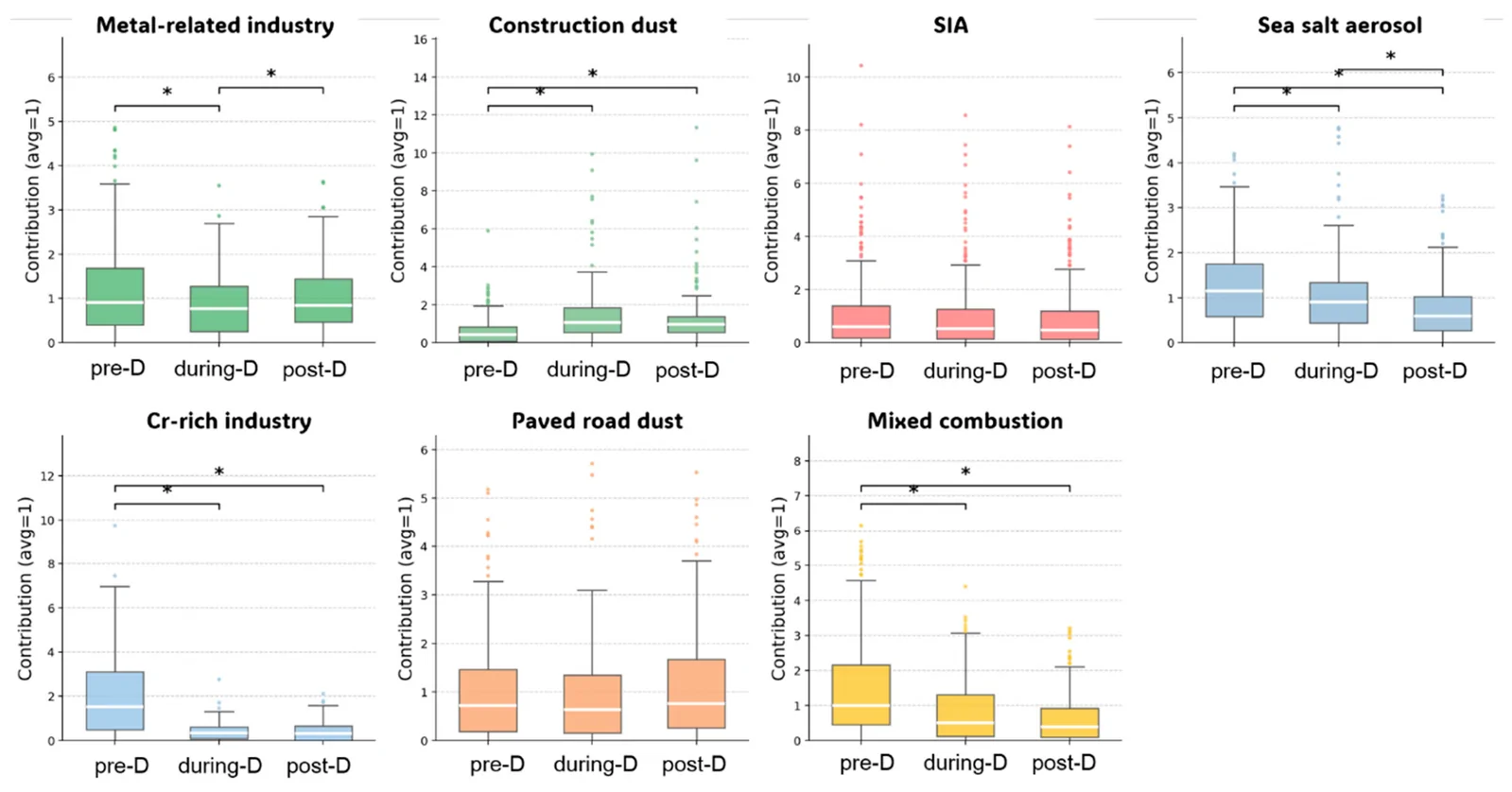
Boxplots of PMF factor contribution scores across the pre-D, during-D, and post-D periods. Note: Kruskal–Wallis test with Dunn’s post hoc test. Significance levels: * *p* < 0.05; ns, not significant.

The distributions of source contributions were compared across the pre-D, during-D, and post-D periods (Figure 5). Some sources showed temporal variation patterns, whereas others did not, indicating that PM_2.5_ source contributions did not vary uniformly across the pre-D, during-D, and post-D periods. In the unadjusted comparison, SIA, which made the largest contribution, and paved road dust, which accounted for 11.1% of total PM_2.5_, did not show significant differences among the three periods (*p* > 0.05). In contrast, the remaining five sources showed significant temporal differences in their contribution distributions (*p* < 0.05).

SIA remained the dominant contributor to PM_2.5_ across all three periods, with no statistically significant difference in its contribution among pre-D, during-D, and post-D (*p* > 0.05). This result is consistent with the observation that SOR and NOR showed no substantial changes across periods, suggesting that the oxidation efficiency and gas-to-particle conversion processes driving SIA formation were relatively stable despite the overall reduction in PM_2.5_ mass concentration. SIA is primarily formed through atmospheric oxidation and gas-to-particle conversion of precursor gases, including SO_2_, NO_x_, and NH_3_, producing sulfate, nitrate, and ammonium aerosols, all of which were identified as dominant markers of the SIA factor in this study [63,64,65,66]. Therefore, the persistent contribution of SIA suggests that secondary aerosol formation was not simply reduced in proportion to social distancing-related restrictions on social and economic activities. This may be because SIA formation is governed not only by precursor emissions but also by atmospheric oxidation capacity, gas-particle partitioning, meteorological conditions, and regional transport. Furthermore, some precursor emissions may not have decreased substantially during the during-D period. For example, traffic volume data from the Seoul metropolitan area showed that passenger car traffic decreased by only 1.7% in 2020 despite social distance-related restrictions [67], suggesting that vehicle-derived NO_x_ and NH_3_ emissions remained largely unchanged. Overall, the sustained SIA contribution likely reflects the combined effects of relatively persistent precursor emissions and favorable atmospheric conditions for secondary aerosol formation. A similar pattern was observed for paved road dust, which also showed no significant difference across the three periods (*p* > 0.05), likely reflecting the continued level of vehicle traffic on roads throughout the social-distancing period.

In contrast, the remaining five sources showed significant differences among the social distancing-related periods, and the results of the post hoc tests indicated source-specific variation patterns. The metal-related industry showed a significant temporal decrease during the during-D period compared with the pre-D period, followed by a significant increase during the post-D period (*p* < 0.05). This temporal pattern coincided with broader changes in industrial activity over the same period. Industrial facilities involved in metal processing and manufacturing are known to emit characteristic metal species such as Cr, Fe, and Mn [62,68]. The subsequent increase observed during the post-D period was also temporally consistent with the broader resumption of industrial activity.

Construction dust showed significantly higher contributions during the during-D and post-D periods than during pre-D (*p* < 0.05), with no significant difference between during-D and post-D. This pattern indicates that construction dust increased after the onset of social distancing and remained elevated during post-D. This pattern is consistent with continued construction and civil engineering activities or local construction-related fugitive-dust influences during the during-D and post-D periods. In Korea, construction orders reached 287 trillion Korean won (KRW) in 2020, representing a 12.2% increase from the previous year, and domestic construction orders also increased by 8% year-on-year [69]. In addition, the Seoul Metropolitan Government implemented investments worth KRW 2.6 trillion by 2022 as part of the Green New Deal, including measures to respond to the COVID-19 economic crisis [70]. These external indicators provide contextual evidence that construction and infrastructure activities remained substantial during the study period. The higher PMF-derived construction-dust contributions observed during the during-D and post-D periods are therefore consistent with the possibility that local construction, infrastructure development, and surface-disturbance activities remained active during these periods.

Sea-salt aerosol showed significant differences among the three periods (*p* < 0.05), with contributions generally decreasing from the pre-D to the during-D and post-D periods. Because the sea-salt factor in the integrated PMF solution was characterized primarily by a strong Na^+^ signature, its temporal variation is likely associated with changes in marine aerosol transport, air-mass pathways, and meteorological conditions rather than direct changes in local anthropogenic emissions [65]. Backward-trajectory analysis of the selected days with relatively high sea-salt contributions showed that air masses generally approached the monitoring site from the western sector, spanning northwestern to southwestern pathways (Supplementary Note S3; Tables S12 and S17). Among the 15 selected cases, 14 trajectories passed over the Yellow Sea or adjacent marine areas west of the Korean Peninsula before reaching Seoul (Figures S1 and S5). This pattern supports the potential influence of regional marine aerosol transport on the resolved sea-salt factor. Thus, the decrease in sea-salt aerosol contribution after the onset of social distancing may reflect differences in regional transport conditions or atmospheric circulation patterns affecting marine aerosol inflow, rather than the direct effect of social distancing-related activity restrictions.

The Cr-rich industrial factor showed significantly lower contributions during the during-D and post-D periods than during the pre-D period (*p* < 0.05). This temporal pattern was consistent with the measured Cr concentration, which decreased from 4.83 ng/m^3^ during the pre-D period to 1.40 and 1.15 ng/m^3^ during the during-D and post-D periods, respectively. This decrease is interpreted as a reduction in the receptor-based contribution of the Cr-rich factor rather than as direct evidence of reduced Cr emissions from specific industrial facilities.

The mixed-combustion factor also showed significantly lower contributions during the during-D and post-D periods than during the pre-D period (*p* < 0.05). This factor was characterized by Cl^−^, K^+^, OC, EC, and trace metals, reflecting the combined influence of urban combustion activities, fuel combustion, heating, cooking emissions, and combustion-related industrial processes [63,64]. Therefore, the reduction in socioeconomic activities and fuel combustion during the during-D period may have contributed to the decrease in this source contribution. Although this factor contributed less than 1% to total PM_2.5_ mass, its significant temporal change suggests that pandemic-related changes in activity patterns were reflected in specific combustion-related marker species.

Overall, the temporal comparison of source contributions showed that the PM_2.5_ sources did not follow a simple uniform reduction-or-recovery pattern across the pre-D, during-D, and post-D periods. While metal-related industrial activity temporarily decreased during the during-D period and partially rebounded during the post-D period, Cr-rich industry and mixed combustion remained lower after the onset of social distancing. In the unadjusted comparison, SIA and paved road dust showed no significant temporal differences, whereas construction dust remained elevated during the during-D and post-D periods. These contrasting responses likely reflect differences in the processes controlling individual sources: SIA is influenced by precursor availability and atmospheric transformation, paved road dust by continued traffic and resuspension, and construction dust by ongoing construction, infrastructure development, and surface-disturbance activities. Thus, the observed source contributions did not vary proportionally with the broader changes in social activity across the three periods, indicating source-specific temporal restructuring rather than a simple return to the pre-D pattern.

#### 3.3.3. Meteorological Characteristics and Meteorology-Adjusted Period Effects

Meteorological conditions differed across the pre-D, during-D, and post-D periods (Table S18). Daily mean temperature increased from 11.8 ± 9.4 °C during the pre-D period to 12.7 ± 10.6 °C during the during-D period and 14.0 ± 10.0 °C during the post-D period (Kruskal–Wallis, *p* < 0.05). Relative humidity also increased across the three periods (56.7 ± 13.9%, 64.0 ± 13.6%, and 68.1 ± 13.5%, respectively; *p* < 0.05), while mean wind speed was higher during the during-D and post-D periods than during the pre-D period (1.89 ± 0.66, 2.28 ± 0.64, and 2.25 ± 0.66 m/s, respectively; *p* < 0.05). Precipitation also differed among the three periods (*p* < 0.05), whereas solar radiation showed no significant temporal difference (*p* = 0.68). These results demonstrate that meteorological conditions varied across the study periods and therefore warranted adjustment when evaluating period-specific differences in PM_2.5_ and related indicators.

After adjustment for temperature, relative humidity, wind speed, precipitation, solar radiation, and seasonal variation using GAMs, PM_2.5_ concentrations remained significantly lower during both the during-D and post-D periods relative to the pre-D period (Table S19). The adjusted PM_2.5_ concentrations were 13.8% lower during the during-D period (95% CI: −22.6% to −3.9%; *p* < 0.05) and 19.7% lower during the post-D period (95% CI: −28.1% to −10.2%; *p* < 0.05). No significant difference was observed between the during-D and post-D periods (−6.8%; *p* = 0.18). For the secondary aerosol indicators, NOR remained non-significantly different among the three periods after meteorological adjustment. In contrast, SOR, which showed no significant period difference in the unadjusted comparison, was significantly lower during both the during-D (β = −0.173, *p* < 0.05) and post-D periods (β = −0.168, *p* < 0.05) relative to the pre-D period, with no significant difference between the during-D and post-D periods (*p* = 0.92). The emergence of these SOR differences after adjustment suggests that inter-period meteorological variability may have masked period-specific differences in sulfur oxidation in the unadjusted comparison.

Meteorological adjustment generally preserved the source-specific temporal patterns identified from the PMF analysis. SIA showed no significant period effects after adjustment (during-D vs. pre-D: *p* = 0.68; post-D vs. pre-D: *p* = 0.63). In contrast, metal-related industry remained significantly lower during both the during-D (β = −0.281, *p* < 0.05) and post-D periods (β = −0.145, *p* < 0.05) relative to the pre-D period; however, its contribution was significantly higher during the post-D than during-D period (β = 0.136, *p* < 0.05), indicating a partial rebound after the during-D decrease. Sea-salt aerosol decreased during both the during-D (β = −0.320, *p* < 0.05) and post-D periods (β = −0.598, *p* < 0.05), with a further significant decrease from the during-D to post-D period (β = −0.277, *p* < 0.05). Paved road dust showed no significant difference between the during-D and pre-D periods (*p* = 0.64), but was significantly higher during the post-D period than during both the pre-D (β = 0.183, *p* < 0.05) and during-D periods (β = 0.224, *p* < 0.05). Construction dust remained significantly higher during both the during-D (β = 0.889, *p* < 0.05) and post-D periods (β = 0.777, *p* < 0.05) relative to the pre-D period, with no significant difference between the during-D and post-D periods (*p* = 0.24). Cr-rich industry remained significantly lower during both periods relative to the pre-D period (both *p* < 0.05), with no significant difference between the during-D and post-D periods. Mixed combustion also remained significantly lower during the during-D (β = −0.277, *p* < 0.05) and post-D periods (β = −0.422, *p* < 0.05), with a further decrease during the post-D relative to the during-D period (β = −0.145, *p* < 0.05). Overall, the meteorology-adjusted results indicate that most of the source-specific temporal patterns observed in the unadjusted analyses persisted after accounting for meteorological and seasonal variability.

Sensitivity analyses using scaled-t robust GAMs for construction dust and SIA yielded the same significance patterns as the primary Gaussian GAMs: construction dust remained significantly higher during both the during-D and post-D periods relative to the pre-D period, whereas SIA showed no significant period effects (Table S20).

### 3.4. Study Limitations

This study has several limitations. First, the analysis was based on a single urban monitoring site in Gwangjin-gu and therefore does not capture the spatial heterogeneity of PM_2.5_ sources across Seoul. Differences in traffic intensity, construction activity, industrial influence, land use, and local environmental conditions may result in different source profiles and temporal patterns at other locations. Accordingly, the findings should be interpreted as site-specific and require confirmation through multi-site analyses. In addition, sampling was conducted primarily on weekdays as part of the routine monitoring program. Therefore, differences between weekday and weekend traffic and activity patterns were not captured, and traffic- or activity-related source contributions should be interpreted as representative primarily of weekday conditions.

Second, uncertainties inherent in the analytical approaches should be considered. PMF results, particularly for minor trace-metal-related factors, may be sensitive to uncertainty specification and factor resolution, although the retained trace-metal species showed adequate signal-to-noise characteristics. Error-estimation diagnostics supported the reproducibility of the low-contribution factors emphasized in this study. In the pre-D seven-factor solution, bootstrap mapping rates were 99%, 100%, and 97% for the metal-related industrial, Cr-rich industrial, and mixed-combustion factors, respectively, and the DISP analysis showed no factor swaps at any tested dQmax level (4, 8, 15, or 25). However, the construction-dust factor showed a lower bootstrap mapping rate (48%), indicating comparatively greater uncertainty in its source attribution. In addition, although temporal correlations supported the correspondence of several matched factors, absolute source-contribution fractions differed between the pre-D-only and integrated PMF solutions, and the correspondence for construction dust was comparatively weak. Therefore, temporal changes were interpreted primarily within the integrated PMF solution, while absolute source contributions and the construction-dust factor in particular should be interpreted with caution. OCsec was estimated using the EC-tracer method and depended on the assumed primary OC/EC reference ratio. The sensitivity analysis showed that its absolute magnitude varied with the selected reference ratio, particularly during the pre-D period; therefore, OCsec should be interpreted as a semi-quantitative indicator rather than an absolute measurement. The HYSPLIT analysis was also restricted to selected high sea-salt-contribution cases and thus provides supporting evidence for marine transport but does not quantify period-level changes in the frequency of regional transport pathways.

Finally, the study was observational and cannot establish direct causal effects of social-distancing policies. Although measured meteorological variability and seasonal patterns were accounted for using GAMs, residual confounding from unmeasured or incompletely represented factors, including boundary-layer dynamics, synoptic-scale circulation, regional transport, socioeconomic activity, and local development, cannot be excluded. In addition, South Korea implemented long-term air-quality policies during the study period, including the Comprehensive Plan for Fine Dust Management (2020–2024), which may also have contributed to changes in PM_2.5_ concentrations and source contributions independently of social-distancing measures [71]. These concurrent policy effects could not be fully separated from the study-period effects in the present observational design. Therefore, the observed differences should be interpreted as meteorology-adjusted temporal associations and plausible contextual patterns rather than as evidence of direct policy-driven effects.

## 4. Conclusions

This six-year study at an urban monitoring site in Gwangjin-gu showed that PM_2.5_ mass concentration, chemical composition, and PMF-derived source contributions followed source-specific temporal trajectories across the pre-D, during-D, and post-D periods rather than a uniform reduction-and-recovery pattern. PM_2.5_ concentrations remained significantly lower during the during-D and post-D periods than during the pre-D period after adjustment for meteorological and seasonal variability. Among the resolved sources, SIA remained temporally stable after meteorological adjustment, whereas metal-related industry showed a decrease during the during-D period followed by a partial rebound during the post-D period. Construction dust remained elevated during both the during-D and post-D periods, while paved road dust showed a significant post-D increase after meteorological adjustment. Cr-rich industry and mixed combustion remained lower than during the pre-D period. These findings indicate that changes in total PM_2.5_ mass alone do not fully represent concurrent changes in source composition and that the observed patterns should be interpreted as site-specific temporal associations rather than as direct causal effects of social-distancing policies.

From an air-quality management perspective, the persistence of SIA indicates that further reductions in PM_2.5_ require continued control of gaseous precursors involved in secondary aerosol formation, in addition to direct particulate-emission control. The post-D increase in paved road dust and the sustained elevation of construction dust further highlight the importance of managing non-exhaust traffic emissions, road-surface resuspension, and construction-related fugitive dust, while the temporal variability of industrial factors supports continued monitoring and control of industrial trace-metal emissions. Overall, the results support a source-oriented management framework in which secondary aerosol formation, non-exhaust traffic sources, construction activities, and industrial emissions are addressed separately. Multi-site analyses incorporating comparable meteorological adjustment are needed to determine whether these source-specific patterns are reproducible across Seoul.

## Figures and Tables

**Table 1:** Concentration of PM_2.5_ across pre-D, during-D, and post-D periods (μg/m^3^).

Period	N	Mean *	S.D.	Max	*p*-Value
Pre-D	349	25.54 a	18.06	140.41	<0.05
During-D	262	20.91 b	15.79	92.65	
Post-D	254	19.28 b	13.74	82.00	

* Different superscripts indicate statistically significant differences among periods based on the Kruskal–Wallis test followed by Dunn’s post hoc test (*p* < 0.05).

**Table 2:** Concentrations of chemical compounds across the periods.

Compounds	Unit	Pre-D		During-D		Post-D		*p*-Value *
		Mean	S.D.	Mean	S.D.	Mean	S.D.	
Cl^−^	μg/m^3^	0.48 a	0.36	0.30 b	0.27	0.27 b	0.26	<0.05
NO_3_^−^		5.81 a	6.47	5.42 a	6.91	5.00 a	6.30	0.33
SO_4_^2−^		3.66 a	2.99	2.89 b	2.23	2.69 b	1.92	<0.05
Na^+^		0.09 a	0.07	0.08 b	0.06	0.07 b	0.05	<0.05
NH_4_^+^		3.04 a	2.96	2.43 b	2.63	2.30 b	2.50	<0.05
K^+^		0.25 a	0.19	0.20 b	0.13	0.13 c	0.13	<0.05
Ca^2+^		0.02 a	0.04	0.08 b	0.07	0.09 b	0.07	<0.05
Mg^2+^		0.01 a	0.03	0.02 b	0.03	0.04 c	0.03	<0.05
OC		3.83 a	1.87	3.35 b	1.67	3.08 b	1.70	<0.05
EC		0.95 a	0.43	0.47 b	0.20	0.48 b	0.20	<0.05
Al	ng/m^3^	78.30 a	79.94	45.22 b	51.95	48.36 b	38.15	<0.05
Fe		147.90 a	86.72	127.49 b	84.67	122.10 b	72.87	<0.05
Mn		7.79 a	5.12	7.60 a	5.28	7.53 a	5.14	0.81
Cu		8.49 a	6.18	6.62 b	4.88	6.91 b	5.94	<0.05
Pb		15.71 a	12.66	11.37 b	8.15	12.93 b	10.75	<0.05
As		3.17 a	2.59	3.15 a	3.59	5.26 c	6.46	<0.05
Cd		0.60 a	0.69	0.41 b	0.30	0.41 b	0.33	<0.05
Cr		4.83 a	4.00	1.40 b	1.92	1.15 b	1.16	<0.05
Ni		1.54 a	1.36	1.18 b	1.39	1.24 a	1.94	<0.05

* *p*-values from the Kruskal–Wallis test for differences in concentrations among the three periods. Different lowercase letters (a, b, c) within a row indicate statistically significant differences among periods (Dunn’s post hoc test, *p* < 0.05).

## Data Availability

The PM_2.5_ mass and chemical composition data analyzed in this study were provided by the Seoul Research Institute of Public Health and Environment. The processed data supporting the findings of this study are available from the corresponding author upon reasonable request, subject to approval by the data-providing institution.

## Supplementary Materials

The following supporting information can be downloaded at: https://www.mdpi.com/article/10.3390/toxics14100886/s1, Supplementary Notes S1: Sampling and gravimetric determination of PM_2.5_ mass concentration; Supplementary Notes S2: Analysis and quality assurance and quality control (QA/QC) for chemical species; Supplementary Notes S3: Backward-trajectory analysis; Table S1: Instrumental and analytical conditions for IC analysis; Table S2: Instrumental and analytical conditions for ED-XRF analysis; Table S3: Classification of study periods based on the social distancing policy in South Korea; Table S4: Season-specific 5th-percentile OC/EC ratios used as primary reference values for OCsec estimation; Table S5: Regression statistics for the relationships between EC and OCtot or OCsec across the three study periods; Table S6: Sensitivity of period-specific OCsec estimates to alternative season-specific OC/EC reference ratios; Table S7: Sensitivity of EC–OCsec relationships to alternative season-specific OC/EC reference ratios; Table S8: Period-specific mean concentrations of SO_2_, SO_4_^2−^, NO_2_, and NO_3_^−^; and corresponding SOR and NOR values; Table S9: Varimax-rotated component matrix from PCA using the pre-D dataset; Table S10: Statistics of input data for PMF using the pre-D dataset; Table S11: Results of PMF analysis using the pre-D dataset; Table S12: Characteristics of the five pre-distancing sampling days with the highest PMF-derived sea-salt factor contribution scores selected for HYSPLIT backward-trajectory analysis; Table S13: Error-estimation diagnostics for the selected seven-factor PMF solution; Table S14: Statistics of input data for PMF using total dataset; Table S15: Results of PMF analysis using total dataset; Table S16: Comparison of pre-D-only PMF source contributions with integrated PMF contributions recovered for the same pre-D period; Table S17: Characteristics of high sea-salt contribution days selected for HYSPLIT backward-trajectory analysis during the during-distancing and post-distancing periods; Table S18: Meteorological conditions during the pre-D, during-D, and post-D periods; Table S19: Meteorology-adjusted GAM results for PM_2.5_, SOR, NOR, and the seven PMF-derived source contribution scores; Table S20: Sensitivity analysis comparing Gaussian and scat robust GAMs for construction dust and secondary inorganic aerosol (SIA); Figure S1: Seventy-two-hour backward air-mass trajectories for the five pre-distancing sampling days with the highest PMF-derived sea-salt factor contribution scores; Figure S2: Heatmap for Pearson correlation coefficients between Step 1 (pre-D-only) and Step 2 (integrated) PMF factor contribution time series, overlapping period (2018–2020); Figure S3: Source profiles and time-series contributions of the PM_2.5_ factors resolved by PMF using total dataset; Figure S4: Temporal variations in PMF source contributions across pre-, during-, and post-D periods (2018–2024); Figure S5: Seventy-two-hour backward air-mass trajectories for the five sampling days with the highest PMF-derived sea-salt factor contribution scores during the (a) during-distancing and (b) post-distancing periods.
