# Supplementary material for: Restructuring of PM2.5 Composition and Source Contributions Across COVID-19 Social Distancing Periods: A Case Study at an Urban Monitoring Site in Seoul, South Korea
Source: Toxics. 2026 Oct 1;14(10):886. doi: 10.3390/toxics14100886 (PMC13709736; doi:10.3390/toxics14100886)
Supplement: Supplementary file 1 [file toxics-14-00886-s001.zip › toxics-4541744-supplementary.pdf]

Supplementary Materials

Supplementary Note S1. Sampling and gravimetric determination of $PM_{2.5}$ mass concentration

$PM_{2.5}$ samples were collected from February 2018 to March 2024 at monitoring site in Gwang-Jin using the low-volume air sampler method. Sampling and chemical analyses were conducted in accordance with the national air quality monitoring network operation guidelines, including the standard procedures for $PM_{2.5}$ manual sampling and chemical speciation analysis. Samples were collected approximately 12 times per month on average, covering various weekdays from Monday to Friday to reflect temporal variability in $PM_{2.5}$ concentrations during routine monitoring periods.

Before sampling, filters were conditioned under controlled temperature and relative humidity conditions and weighed using a microbalance. $PM_{2.5}$ samples were collected using a low-volume air sampler operated at a nominal flow rate of 16.7 L/min. After sampling, the filters were reconditioned under the same conditions and weighed again. $PM_{2.5}$ mass concentration was determined gravimetrically from the difference between pre- and post-sampling filter weights, with correction for field blanks, divided by the total sampled air volume. The $PM_{2.5}$ mass concentration was calculated as follows:

$$PM_{2.5} = \frac{(W_f - W_i) - (W_{FBf} - W_{FBi})}{V_a} \quad \textbf{Eq. S1}$$

where $PM_{2.5}$ is the $PM_{2.5}$ mass concentration $(\mu g/m^3)$, W_i and W_f are the initial and final weights of the sample filter (μg), respectively, W_{FBi} and W_{FBf} are the initial and final weights of the field blank filter (μg), respectively, and V_a is the total sampled air volume (m^3). To ensure valid gravimetric measurement, the effective sampling duration was required to be at least 1080 min, corresponding to 18 h; otherwise, the sample was recollected and reweighed according to the monitoring guideline. After gravimetric analysis, the collected filters were stored in sealed containers under refrigerated or frozen conditions until chemical analysis to minimize contamination and loss of semi-volatile components.

Supplementary Note S2. Analysis and quality assurance and quality control (QA/QC) for chemical species

Analysis of ionic species

Eight ionic species, including Cl^-, NO_3^-, SO_4^{2-}, Na^+, NH_4^+, K^+, Ca^{2+}, and Mg^{2+}, were analyzed. A portion of each $PM_{2.5}$-loaded filter (PTFE 2.0 μm, 47 mm) was extracted with ultrapure water to dissolve water-soluble ionic components. The extract was filtered or centrifuged to remove particulate residues and then analyzed using ion chromatography (IC). The detailed analytical conditions for the ionic species are summarized in **Table S1**.

Table S1. Instrumental and analytical conditions for IC analysis.

Parameter	Anions	Cations
Separation column	Dionex AS-15, 3 × 150 mm	Dionex AS-12, 3 × 150 mm
Guard column	Dionex AG-15, 3 × 30 mm	Dionex AG-12, 3 × 30 mm
Suppressor	ASRS-300, 2 mm	CSRS-300, 2 mm
Detector	CD 20 conductivity detector	CD 20 conductivity detector
Eluent	KOH gradient	MSA mM
Flow rate	0.5 mL/min	0.5 mL/min

Anionic species, including Cl^-, NO_3^-, and SO_4^{2-}, and cationic species, including Na^+, NH_4^+, K^+, Ca^{2+}, and Mg^{2+}, were quantified using calibration curves prepared from standard solutions. Analytical quality was verified using standard solutions, blanks, duplicate samples, and calibration check standards to assess instrumental stability, reproducibility, and potential contamination. The atmospheric concentration of each ionic species was calculated as follows:

$$C = \frac{(X_1 - X_2) \times S}{F} \quad \textbf{Eq. S2}$$

where C is the atmospheric concentration of each ionic species $(\mu g/m^3)$, X_1 is the ionic species concentration in the sample extract $(\mu g/mL)$, X_2 is the ionic species concentration in the blank extract $(\mu g/mL)$, S is the extraction volume (mL), and F is the total sampled air volume (m^3).

QA/QC for ionic species analysis

QA/QC procedures were applied to evaluate the reliability of ionic species measurements. Linearity, method detection limit (MDL), precision, and accuracy were evaluated for each target ion, including Na^+, NH_4^+, K^+, Ca^{2+}, Mg^{2+}, Cl^-, NO_3^-, and SO_4^{2-}.

The linearity of the calibration curves satisfied the acceptance criterion of $R^2 \geq 0.999$ for all ionic species throughout the entire measurement period. The MDLs ranged from 0.001 mg/L (NO_3^-) to 0.031 mg/L (Mg^{2+}), depending on the target ion and analytical year. Analytical precision, expressed as relative standard deviation (% RSD), was within the acceptance criterion of 10% for all ions. Analytical accuracy also satisfied the acceptance range of 75%-125% for all target ions. These results indicate that the ion chromatography analysis met the internal QA/QC criteria for the determination of ionic species in $PM_{2.5}$ samples.

Analysis of carbon

Organic carbon (OC) and elemental carbon (EC) were analyzed using $PM_{2.5}$-loaded quartz-fiber filters (47 mm). The carbonaceous components were determined by a thermal-optical carbon analysis method following the NIOSH Method 5040, in which carbon fractions were separated according to their thermal and optical properties. Briefly, OC was volatilized under an inert atmosphere through stepwise heating, followed by the oxidation of EC under an oxidizing atmosphere.

The amount of carbon evolved at each temperature step was quantified using a carbon analyzer, and the split point between OC and EC was determined by optical correction to account for pyrolyzed organic carbon formed during the thermal process. The concentrations of OC and EC in ambient air were calculated by dividing the measured carbon mass by the total sampled air volume. The atmospheric concentrations of carbonaceous species were calculated as follows:

$$C = \frac{(X_1 - X_2) \times S}{F} \quad \textbf{Eq. S3}$$

where C is the atmospheric concentration of OC or EC $(\mu g/m^3)$, M_s is the carbon mass measured in the sample filter (μg), M_b is the carbon mass measured in the blank filter (μg), and F is the total sampled air volume (m^3).

QA/QC for carbon analysis

QA/QC procedures were applied to evaluate the reliability of carbonaceous species measurements. Linearity, MDL, precision, and accuracy were evaluated for each target carbon, including OC.

The linearity of the calibration curves satisfied the acceptance criterion of $R^2 \geq 0.99$ throughout the entire measurement period. The MDL (OC) was 0.085 µg–0.124 µg. RSD was 0.35%–0.66%, satisfying the acceptance criterion of $\leq$ 10%. Bias was 1.24%–2.69%, satisfying

the acceptance criterion of <5%. These results indicate that the carbon analysis met the internal QA/QC criteria for the determination of OC and EC in $PM_{2.5}$ samples.

Analysis of elemental species

Elemental species, including Be, Mg, Al, Cr, Mn, Fe, Ni, Cu, As, Cd, Pb, and Ca, were analyzed using $PM_{2.5}$-loaded PTFE filters (PTFE 2.0 µm, 47 mm). Elemental species were determined using an energy-dispersive X-ray fluorescence analyzer (ED-XRF). The $PM_{2.5}$-loaded filters were analyzed directly without chemical digestion, allowing non-destructive quantification of particle-bound elemental components. The detailed analytical conditions for the elemental species are summarized in **Table S2**.

Table S2. Instrumental and analytical conditions for ED-XRF analysis.

Parameter		Values
X-ray	Detector	Ge (germanium)
	Windows	8 µm Be
	Energy range	0.7–100 keV
	Resolution	< 140 eV
	Cooling	Liquid nitrogen cooling
X-ray tube	Type	Side window tube
	Anode	Gd
	Rating	25–100 kV, 0.5–24 Ma
	Max power	600 W
	Cooling	Internal water cooling
Stabilization conditions	Temperature	≤ 35°C
	Generator	≤100.0 kV, ≤6.0 mA
	Vacuum	≤0.5 Pa
	Water flow	1.6 L/min
	Water temperature	30 °C

In ED-XRF analysis, each filter sample was irradiated with X-rays, and the characteristic fluorescent X-rays emitted from the target elements were detected and quantified. Elemental concentrations were calculated using calibration curves or instrument-specific calibration factors prepared from standard reference materials or thin-film standards. Blank correction was applied using field or laboratory blank filters to account for potential background signals from filters and handling procedures. The atmospheric concentration of each elemental species was calculated according to the national monitoring guideline as follows:

$C = \frac{(C_s - C_{bk}) \times A_U}{V_a}$ **Eq. S4**

where C is the atmospheric concentration of each elemental species (ng/m^3), C_s is the measured concentration or mass loading of the target element in the sample filter, C_{bk} is the corresponding blank value, A_U is the effective area of the filter used for sample collection, and V_a is the total sampled air volume (m^3).

QA/QC for elemental species analysis

QA/QC procedures were applied to evaluate the reliability of elemental species measurements. Precision, accuracy, and MDL were evaluated for each target element, including Al, As, Be, Ca, Cd, Cr, Cu, Fe, Mg, Mn, Ni, and Pb.

RSD ranged from 0.27% to 2.25% throughout the entire measurement period. These values were within the acceptable range, indicating good analytical repeatability. Analytical accuracy ranged from 97.07% to 99.42% throughout the entire measurement period, satisfying the internal QA/QC criteria. The MDLs ranged from 0.43 ng/mL to 6.34 ng/mL throughout the entire measurement period, depending on the target element.

These results indicate that the ED-XRF analysis met the internal QA/QC criteria for the determination of elemental species in $PM_{2.5}$ samples.

Supplementary Note S3. Backward-trajectory analysis

To examine the potential influence of regional air-mass transport on the sea-salt aerosol factor, backward-trajectory analysis was performed using the Hybrid Single-Particle Lagrangian Integrated Trajectory (HYSPLIT) model provided by the NOAA Air Resources Laboratory through the READY system. For each study period (pre-D, during-D, and post-D), the five sampling days with the highest PMF-derived sea-salt factor contribution scores were selected, resulting in a total of 15 trajectory cases (Table S12 and S17). Seventy-two-hour backward trajectories arriving at the Gwangjin monitoring site (37.5470° N, 127.0925° E) were calculated at an arrival height of 500 m above ground level (AGL) using GDAS1 archived meteorological data and the model vertical velocity option. To ensure consistency across sampling days, the arrival time was fixed at 00:00 UTC (09:00 KST) for all trajectories. The resulting trajectories were qualitatively examined to identify the predominant air-mass approach directions and whether the transport pathways passed over the Yellow Sea or adjacent marine areas west of the Korean Peninsula before reaching Seoul.

Table S3. Classification of study periods based on the social distancing policy in South Korea.

Period	Stage	Date range	Representative restrictions
Pre-distance (pre-D)	0	1 Feb 2018 to 5 May 2020	No COVID-19 social-distancing restrictions
Social distancing (during D)	1-4	6 May 2020 to 17 Apr 2022	Restrictions on private gatherings, business operating hours and capacity, large events, religious gatherings, and selected high-risk facilities
Post-distancing (post-D)	0	18 Apr 2022 to 26 Mar 2024	Most social-distancing restrictions lifted

Table S4. Season-specific 5th-percentile OC/EC ratios used as primary reference values for OCsec estimation.

Season	N	(OC/EC)pri
Spring	225	2.65
Summer	180	2.54
Autumn	224	2.54
Winter	236	3.23

Table S5. Regression statistics for the relationships between EC and OC_{tot} or OC_{sec} across the three study periods.

Period	OC_{tot} or OC_{sec}	Slope	Intercept	Pearson r*
Pre-D	OC_{tot}	2.70	1.28	0.63*
	OC_{sec}	0.19	1.11	0.06
During-D	OC_{tot}	5.99	0.54	0.73*
	OC_{sec}	3.04	0.61	0.47*
Post-D	OC_{tot}	6.09	0.18	0.70*
	OC_{sec}	3.33	0.19	0.49*

* $p<0.05$

Table S6. Sensitivity of period-specific OCsec estimates to alternative season-specific OC/EC reference ratios.

Period	Scenario	Mean ± SD (μg/m³)	Change vs. primary (%)[1]
Pre-D	Minimum	2.664 ± 1.574	+107.6
	5th percentile (Primary)	1.283 ± 1.261	Reference
	10th percentile	1.011 ± 1.191	−21.2
During-D	Minimum	2.778 ± 1.497	+36.5
	5th percentile (Primary)	2.035 ± 1.300	Reference
	10th percentile	1.861 ± 1.265	−8.6
Post-D	Minimum	2.509 ± 1.537	+41.1
	5th percentile (Primary)	1.778 ± 1.339	Reference
	10th percentile	1.605 ± 1.300	−9.7

[1]The season-specific 5th-percentile OC/EC ratio was used as the primary reference scenario.

Table S7. Sensitivity of EC–OCsec relationships to alternative season-specific OC/EC reference ratios.

Reference scenario	Period	N	Slope	Intercept	Pearson r	p-value
Minimum	Pre-D	349	1.364	1.373	0.38	<0.05
	During-D	262	4.692	0.577	0.64	<0.05
	Post-D	254	4.836	0.199	0.62	<0.05
5th percentile (Primary)	Pre-D	349	0.185	1.108	0.06	0.23
	During-D	262	3.038	0.611	0.47	<0.05
	Post-D	254	3.334	0.185	0.49	<0.05
10th percentile	Pre-D	349	0.022	0.991	0.01	0.88
	During-D	262	2.691	0.599	0.43	<0.05
	Post-D	254	2.999	0.173	0.45	<0.05

Table S8. Period-specific mean concentrations of SO_2, SO_4^{2-}, NO_2, and NO_3^- and corresponding SOR and NOR values.

Period	SO_2 (ppm)	SO_4^{2-} (μg/m³)	SOR	NO_2 (ppm)	NO_3^- (μg/m³)	NOR
Pre-D	0.00412	3.78	0.16	0.02810	6.05	0.06
During-D	0.00305	2.89	0.17	0.02101	5.42	0.07
Post-D	0.00269	2.69	0.18	0.02109	5.00	0.07

Note) Values represent arithmetic means for each study period; SO_2 and NO_2 were calculated from daily mean concentrations corresponding to the $PM_{2.5}$ sampling dates.

Table S9. Varimax-rotated component matrix from PCA using the pre-D dataset.

**Species**	**PC1**	**PC2**	**PC3**	**PC4**	**PC5**	**PC6**
$PM_{2.5}$	.369	**.690**	.208	-.041	.091	.021
Cl^-	**.686**	.177	.014	.087	.044	.185
NO_3^-	**.522**	**.727**	.037	.172	.110	.187
SO_4^{2-}	.125	**.854**	.053	.193	.081	.095
Na^+	.091	.350	.326	-.028	-.051	**.673**
NH_4^+	.465	**.810**	.036	.188	.102	.168
K^+	**.512**	.296	.211	-.004	.477	-.031
Ca^{2+}	-.041	.103	.128	-.173	**.834**	.084
Mg^{2+}	.078	.041	-.069	-.027	**.868**	-.090
OC	**.740**	.436	.096	.164	.046	.063
EC	**.613**	.237	.176	**.502**	-.063	-.087
Be	-.032	.206	.486	.496	-.124	-.334
Mg	.028	.005	-.013	**.770**	-.146	-.063
Al	**.626**	.375	.348	.332	.004	.261
Cr	**.573**	.203	**.529**	.392	-.067	.147

Species	PC1	PC2	PC3	PC4	PC5	PC6
Mn	.122	.381	.046	**.556**	-.059	.450
Fe	.291	.191	.271	**.603**	.037	.201
Ni	**.745**	.234	.132	-.093	-.050	-.171
Cu	.598	-.083	.031	.270	.095	.292
As	**.836**	.253	.090	-.017	.080	-.026
Cd	.159	.164	**.846**	.063	-.073	.214
Pb	.122	-.044	**.788**	.071	.245	.041
Ca	.369	**.690**	.208	-.041	.091	.021
Eigenvalue	4.785	3.450	2.338	2.288	1.853	1.199
% Variance	21.752	15.681	10.625	10.402	8.424	5.449
Cumulative %	21.752	37.432	48.058	58.459	66.883	72.332

***Bold: |loading| ≥ 0.5**

Table S10. Statistics of input data for PMF using the pre-D dataset.

Species	Category	S/N	Min	25th	Median	75th	Max
$PM_{2.5}$[a]	Weak	9.00	0.50	14.00	21.20	31.78	140.41
Cl^-	Strong	8.67	0.00	0.25	0.36	0.62	1.78
NO_3^-	Strong	9.00	0.03	1.50	3.87	8.03	45.58
SO_4^{2-}	Strong	9.00	0.44	1.72	2.68	4.83	20.18
Na^+	Strong	7.54	0.00	0.05	0.09	0.13	0.37
NH_4^+	Strong	8.97	0.00	1.06	2.27	4.04	21.28
K^+	Strong	8.27	0.00	0.11	0.22	0.35	1.19
Ca^{2+}	Strong	1.33	0.00	0.01	0.01	0.01	0.23
Mg^{2+}	Bad	0.42	0.00	0.01	0.01	0.01	0.34
OC	Strong	8.97	0.83	2.44	3.37	4.85	11.45
EC	Strong	8.60	0.16	0.64	0.89	1.22	2.81
Al	Strong	8.97	0.00	0.03	0.06	0.10	0.84
Cr	Strong	8.51	0.00	0.00	0.00	0.01	0.02
Mn	Strong	8.97	0.00	0.00	0.01	0.01	0.03
Fe	Strong	9.00	0.01	0.09	0.13	0.20	0.71
Ni	Strong	7.60	0.00	0.00	0.00	0.00	0.01
Cu	Strong	8.71	0.00	0.00	0.01	0.01	0.04
As	Strong	7.05	0.00	0.00	0.00	0.00	0.02
Cd	Strong	7.05	0.00	0.00	0.00	0.00	0.01
Pb	Strong	8.90	0.00	0.01	0.01	0.02	0.08
Mg	Strong	8.98	0.00	0.02	0.03	0.04	0.19

Ca	Strong	8.99	0.01	0.06	0.08	0.11	0.46

a $PM_{2.5}$ mass concentration was designated as the **total variable** in EPA PMF.

Table S11. Results of PMF analysis using the pre-D dataset.

Species	Intercept	Slope	SE	R^2	KS-Test	
					Stat	p-value
$PM_{2.5}$	0.89	0.83	9.56	0.72	0.03	0.86
Cl^-	0.09	0.70	0.19	0.63	0.07	0.06
NO_3^-	0.89	0.71	2.16	0.83	0.03	0.89
SO_4^{2-}	0.99	0.56	1.69	0.53	0.06	0.23
Na^+	0.00	0.97	0.02	0.90	0.13	0.00
NH_4^+	0.26	0.85	1.12	0.85	0.12	0.00
K^+	0.00	0.97	0.05	0.94	0.12	0.00
Ca^{2+}	0.01	0.06	0.01	0.13	0.25	0.00
Mg^{2+}	0.01	0.02	0.00	0.05	0.29	0.00
OC	0.34	0.85	0.97	0.74	0.04	0.68
EC	0.08	0.87	0.26	0.68	0.03	0.89
Al	0.04	0.21	0.03	0.31	0.05	0.32
Cr	0.00	0.84	0.00	0.84	0.18	0.00
Mn	0.00	0.75	0.00	0.86	0.04	0.53
Fe	0.03	0.76	0.03	0.83	0.05	0.34
Ni	0.00	0.45	0.00	0.50	0.07	0.09
Cu	0.00	0.56	0.00	0.52	0.07	0.08
As	0.00	0.54	0.00	0.60	0.07	0.10
Cd	0.00	0.26	0.00	0.26	0.06	0.16
Pb	0.00	0.62	0.00	0.73	0.07	0.07
Mg	0.01	0.39	0.01	0.64	0.06	0.23
Ca	0.04	0.49	0.03	0.41	0.06	0.19

Table S12. Characteristics of the five pre-distancing sampling days with the highest PMF-derived sea-salt factor contribution scores selected for HYSPLIT backward-trajectory analysis.

Period	Rank	Sampling date	Season	Contribution score[1]	$PM_{2.5}$ (μg·m⁻³)	Na^{+} (μg·m⁻³)	Dominant air-mass pathway	Yellow Sea passage
Pre-D	1	27 Mar 2019	Spring	4.200	64.00	0.31	W–SW	Yes
	2	4 Mar 2019	Spring	4.146	104.00	0.27	Local/regional recirculation	**No**
	3	14 May 2018	Spring	4.066	52.09	0.26	W–SW	Yes
	4	14 Jan 2019	Winter	3.750	120.87	0.22	W–NW	Yes
	5	16 Sep 2018	Autumn	3.552	24.22	0.17	W–NW	Yes

[1]PMF-derived sea-salt factor contribution scores were normalized to an average value of 1.
Note; For each study period, the five sampling days with the highest sea-salt factor contribution scores were selected. $PM_{2.5}$ and Na^{+} represent the measured concentrations on the corresponding sampling dates. Seasons were classified as spring (March–May), summer (June–August), autumn (September–November), and winter (December–February). Dominant air-mass pathways and Yellow Sea passage were determined from 72 h HYSPLIT backward trajectories arriving at the monitoring site at 00:00 UTC and 500 m AGL.

Table S13. Error-estimation diagnostics for the selected seven-factor PMF solution.

Source	Bootstrap mapping (%)	DISP swaps, dQmax = 4	dQmax = 8	dQmax = 15	dQmax = 25
Cr-rich industrial factor	100	0	0	0	0
Paved road dust	89	0	0	0	0
Construction dust	48	0	0	0	0
SIA	85	0	0	0	0
Sea-salt aerosol	100	0	0	0	0
Metal-related industry	99	0	0	0	0
Mixed combustion	97	0	0	0	0

Table S14. Statistics of input data for PMF using total dataset.

Species	Category	S/N	Min	25th	Median	75th	Max
$PM_{2.5}$[a]	Weak	9.00	0.50	11.37	18.00	28.71	140.41
Cl^-	Strong	8.86	0.00	0.13	0.28	0.48	1.78
NO_3^-	Strong	9.00	0.03	1.15	3.04	7.34	45.58
SO_4^{2-}	Strong	9.00	0.02	1.42	2.36	4.25	20.18
Na^+	Strong	8.09	0.00	0.04	0.07	0.11	0.42
NH_4^+	Strong	8.89	0.00	0.76	1.77	3.55	21.28
K^+	Strong	8.04	0.00	0.08	0.16	0.27	1.24
Ca^{2+}	Strong	3.08	0.00	0.01	0.02	0.07	0.41
Mg^{2+}	Strong	1.35	0.00	0.01	0.01	0.02	0.34
OC	Strong	8.96	0.14	2.20	3.13	4.46	11.45
EC	Strong	8.15	0.08	0.39	0.57	0.85	2.81
Al	Strong	8.85	0.00	0.03	0.04	0.07	0.84
Cr	Strong	7.56	0.00	0.00	0.00	0.00	0.03
Mn	Strong	8.97	0.00	0.00	0.01	0.01	0.03
Fe	Strong	8.98	0.00	0.07	0.12	0.18	0.74
Ni	Strong	7.21	0.00	0.00	0.00	0.00	0.02
Cu	Strong	8.67	0.00	0.00	0.01	0.01	0.08
As	Strong	6.92	0.00	0.00	0.00	0.00	0.04
Cd	Strong	6.53	0.00	0.00	0.00	0.00	0.01
Pb	Strong	8.82	0.00	0.01	0.01	0.02	0.08
Mg	Strong	8.96	0.00	0.02	0.02	0.04	0.21
Ca	Strong	8.91	0.00	0.05	0.07	0.10	0.90

a $PM_{2.5}$ mass concentration was designated as the **total variable** in EPA PMF.

Table S15. Results of PMF analysis using total dataset.

Species	Intercept	Slope	SE	R^2	Stat	p-value
$PM_{2.5}$	3.55	0.64	8.27	0.62	0.06	0.01
Cl^-	0.05	0.72	0.14	0.73	0.07	0.00
NO_3^-	0.58	0.77	1.58	0.91	0.04	0.09
SO_4^{2-}	0.91	0.55	1.33	0.55	0.04	0.21
Na^+	0.02	0.71	0.03	0.71	0.12	0.00
NH_4^+	-0.02	1.00	0.92	0.90	0.14	0.00
K^+	0.05	0.56	0.08	0.54	0.06	0.01
Ca^{2+}	0.01	0.44	0.02	0.55	0.05	0.02
Mg^{2+}	0.01	0.10	0.01	0.11	0.15	0.00
OC	0.26	0.87	1.00	0.72	0.02	0.91
EC	0.09	0.80	0.20	0.73	0.04	0.19
Al	0.03	0.31	0.02	0.41	0.06	0.00
Cr	0.00	0.78	0.00	0.78	0.13	0.00
Mn	0.00	0.65	0.00	0.76	0.03	0.43
Fe	0.04	0.64	0.03	0.72	0.03	0.47
Ni	0.00	0.21	0.00	0.27	0.02	0.68
Cu	0.00	0.49	0.00	0.47	0.06	0.01
As	0.00	0.40	0.00	0.59	0.08	0.00
Cd	0.00	0.34	0.00	0.38	0.04	0.18
Pb	0.00	0.72	0.00	0.83	0.03	0.31
Mg	0.01	0.44	0.01	0.59	0.03	0.31
Ca	0.03	0.56	0.04	0.48	0.04	0.07

Table S16. Comparison of pre-D-only PMF source contributions with integrated PMF contributions recovered for the same pre-D period.

Source	Step 1 factor	Pre-D-only PMF (%)	Step 2 factor	Integrated full-period (%)	Integrated, Pre-D subset (%)	Difference [a] (%p)	r [b]
SIA	F4	56.2	F3	42.5	42.2	+14.0	0.98
Paved road dust	F2	34.2	F6	11.1	10.3	+23.8	0.55
Sea-salt aerosol	F5	6.6	F4	11.0	12.8	−6.1	0.76
Cr-rich industry	F1	3.0	F5	0.0	0.0	+3.0	0.96
Construction dust	F3	0.0	F2	7.4	4.1	−4.1	0.18
Metal-related industry	F6	0.0	F1	28.0	30.6	−30.6	0.90
Mixed combustion	F7	0.0	F7	0.0	0.0	0.0	0.91

a Difference = Pre-D-only PMF (%) − Integrated PMF, Pre-D subset (%). b r = Pearson correlation between the Step 1 (pre-D-only) and Step 2 (integrated) factor contribution time series (avg = 1 units), computed over the 354 overlapping Pre-D sampling dates—the same quantity used for the original factor matching.

Table S17. Characteristics of high sea-salt contribution days selected for HYSPLIT backward-trajectory analysis during the during-distancing and post-distancing periods.

Period	Rank	Sampling date	Season	Contribution score[1]	$PM_{2.5}$ ($\mu g \cdot m^{-3}$)	Na^+ ($\mu g \cdot m^{-3}$)	Dominant air-mass pathway	Yellow Sea passage
During-D	1	21 Feb 2021	Winter	4.781	40.61	0.29	SW	Yes
	2	19 Feb 2021	Winter	4.745	23.78	0.35	NW	Yes
	3	9 Jan 2022	Winter	4.578	92.65	0.26	NW	Yes
	4	13 Aug 2020	Summer	4.435	13.87	0.26	S–SW	Yes
	5	19 Nov 2021	Autumn	3.757	61.57	0.22	SW	Yes
Post-D	1	18 May 2022	Spring	3.266	33.00	0.24	W–SW	Yes
	2	9 Dec 2023	Winter	3.192	30.30	0.23	S–SW	Yes
	3	7 Jan 2023	Winter	3.167	82.00	0.19	W–NW / recirculation	Yes
	4	2 Mar 2024	Spring	3.065	11.87	0.21	NW	Yes
	5	19 Mar 2024	Spring	3.039	24.39	0.17	NW	Yes

[1]PMF-derived sea-salt factor contribution scores were normalized to an average value of 1.

Note: For each study period, the five sampling days with the highest sea-salt factor contribution scores were selected. $PM_{2.5}$ and Na^+ represent the measured concentrations on the corresponding sampling dates. Seasons were classified as spring (March–May), summer (June–August), autumn (September–November), and winter (December–February). Dominant air-mass pathways and Yellow Sea passage were determined from 72 h HYSPLIT backward trajectories arriving at the monitoring site at 00:00 UTC and 500 m AGL.

Table S18. Meteorological conditions during the pre-D, during-D, and post-D periods.

Meteorological variable	Pre-D	During-D	Post-D	p-value[1]
Temperature (°C)	11.8 ± 9.4	12.7 ± 10.6	14.0 ± 10.0	<0.05
Relative humidity (%)	56.7 ± 13.9	64.0 ± 13.6	68.1 ± 13.5	<0.05
Wind speed (m/s)	1.89 ± 0.66	2.28 ± 0.64	2.25 ± 0.66	<0.05
Precipitation (mm)	2.0 ± 7.7	2.3 ± 9.2	5.8 ± 18.9	<0.05
Solar radiation (MJ/m²)	13.9 ± 6.7	14.3 ± 6.5	13.9 ± 7.6	0.68

[1] p-values are from Kruskal–Wallis tests comparing the three study periods.

Table S19. Meteorology-adjusted GAM results for $PM_{2.5}$, SOR, NOR, and the seven PMF-derived source contribution scores.

Outcome	Contrast	Estimate	Scale[1]	95% CI	p-value
$PM_{2.5}$	During-D vs. Pre-D	−13.8%	*% (back-transformed)*	−22.6%, −3.9%	<0.05
	Post-D vs. Pre-D	−19.7%		−28.1%, −10.2%	<0.05
	Post-D vs. During-D	−6.8%		−15.9%, 3.3%	0.18
SOR	During-D vs. Pre-D	−0.173	*β (logit link)*	−0.275, −0.070	<0.05
	Post-D vs. Pre-D	−0.168		−0.271, −0.064	<0.05
	Post-D vs. During-D	0.005		−0.091, 0.101	0.92
NOR	During-D vs. Pre-D	0.084	*β (logit link)*	−0.026, 0.194	0.13
	Post-D vs. Pre-D	0.009		−0.107, 0.125	0.88
	Post-D vs. During-D	−0.075		−0.182, 0.032	0.17
Secondary inorganic aerosol (SIA)	During-D vs. Pre-D	0.041	*β (identity link)*	−0.155, 0.237	0.68
	Post-D vs. Pre-D	−0.050		−0.253, 0.152	0.63
	Post-D vs. During-D	−0.092		−0.282, 0.099	0.35
Metal-related industry	During-D vs. Pre-D	−0.281	*β (identity link)*	−0.408, −0.153	<0.05
	Post-D vs. Pre-D	−0.145		−0.276, −0.013	<0.05
	Post-D vs. During-D	0.136		0.013, 0.259	<0.05
Sea-salt aerosol	During-D vs. Pre-D	−0.320	*β (identity link)*	−0.460, −0.180	<0.05
	Post-D vs. Pre-D	−0.598		−0.743, −0.452	<0.05

Outcome	Contrast	Estimate	Scale[1]	95% CI	p-value
	Post-D vs. During-D	−0.277		−0.413, −0.142	<0.05
Paved road dust	During-D vs. Pre-D	−0.041	*β (identity link)*	−0.213, 0.130	0.64
	Post-D vs. Pre-D	0.183		0.006, 0.360	<0.05
	Post-D vs. During-D	0.224		0.056, 0.392	<0.05
Construction dust	During-D vs. Pre-D	0.889	*β (identity link)*	0.701, 1.077	<0.05
	Post-D vs. Pre-D	0.777		0.581, 0.973	<0.05
	Post-D vs. During-D	−0.111		−0.298, 0.075	0.24
Cr-rich industry	During-D vs. Pre-D	−1.273	*β (identity link)*	−1.462, −1.084	<0.05
	Post-D vs. Pre-D	−1.245		−1.441, −1.049	<0.05
	Post-D vs. During-D	0.028		−0.155, 0.211	0.76
Mixed combustion	During-D vs. Pre-D	−0.277	*β (identity link)*	−0.413, −0.141	<0.05
	Post-D vs. Pre-D	−0.422		−0.562, −0.281	<0.05
	Post-D vs. During-D	−0.145		−0.276, −0.014	<0.05

[1] PM2.5 estimates are back-transformed percentage differences from the log-scale GAM coefficient (Gaussian GAM on log(PM2.5)). SOR and NOR estimates are beta-regression coefficients on the logit scale (mgcv::betar). SIA and the six remaining PMF-derived source scores are identity-link coefficients from Gaussian GAMs on the factor contribution score (avg = 1). All models included Pre-D as the reference category and were adjusted for temperature, relative humidity, wind speed, log(1+precipitation), solar radiation, and a cyclic smooth term for day of year (Post-D vs. During-D contrasts obtained by re-fitting with During-D as the reference category). Deviance explained: PM2.5 = 46.1%, SOR = 39.4%, NOR = 44.7%, SIA = 38.7%, metal-related industry = 38.1%, sea-salt aerosol = 20.9%, paved road dust = 21.3%, construction dust = 23.2%, Cr-rich industry = 38.0%, mixed combustion = 57.8%.

Table S20. Sensitivity analysis comparing Gaussian and scat robust GAMs for construction dust and secondary inorganic aerosol (SIA).

Outcome	Model	Contrast	β	95% CI	p-value
**Construction dust**	Gaussian (primary)	During-D vs. Pre-D	0.889	0.701, 1.077	<0.05
	Gaussian (primary)	Post-D vs. Pre-D	0.777	0.581, 0.973	<0.05
	scat robust (sensitivity)	During-D vs. Pre-D	0.674	0.560, 0.787	<0.05
	scat robust (sensitivity)	Post-D vs. Pre-D	0.652	0.533, 0.770	<0.05
**Secondary inorganic aerosol (SIA)**	Gaussian (primary)	During-D vs. Pre-D	0.041	−0.155, 0.237	0.68
	Gaussian (primary)	Post-D vs. Pre-D	−0.050	−0.253, 0.152	0.63
	scat robust (sensitivity)	During-D vs. Pre-D	−0.024	−0.147, 0.099	0.70
	scat robust (sensitivity)	Post-D vs. Pre-D	−0.036	−0.164, 0.093	0.59

Note: Scaled (t) robust GAMs produced the same significance patterns as the primary Gaussian models for both construction dust and SIA, supporting the robustness of the results. N = 865 for both outcomes.

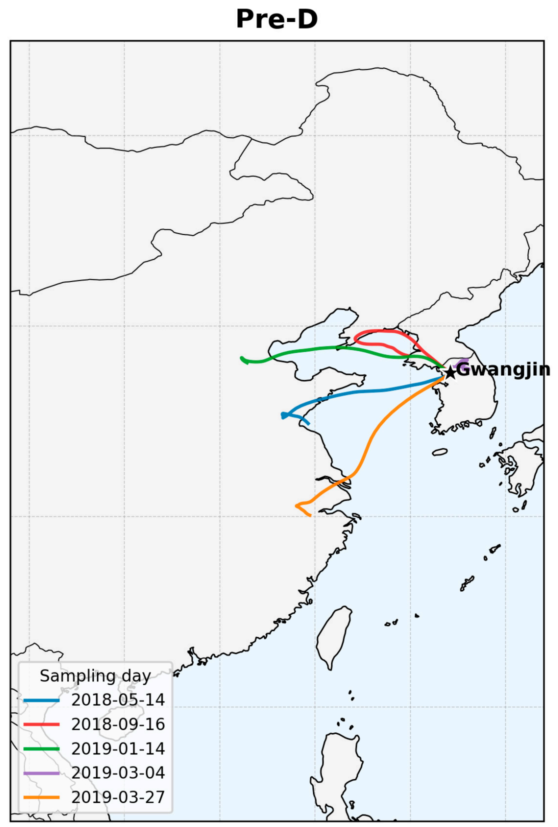

Figure S1: Seventy-two-hour backward air-mass trajectories for the five pre-distancing sampling days with the highest PMF-derived sea-salt factor contribution scores.

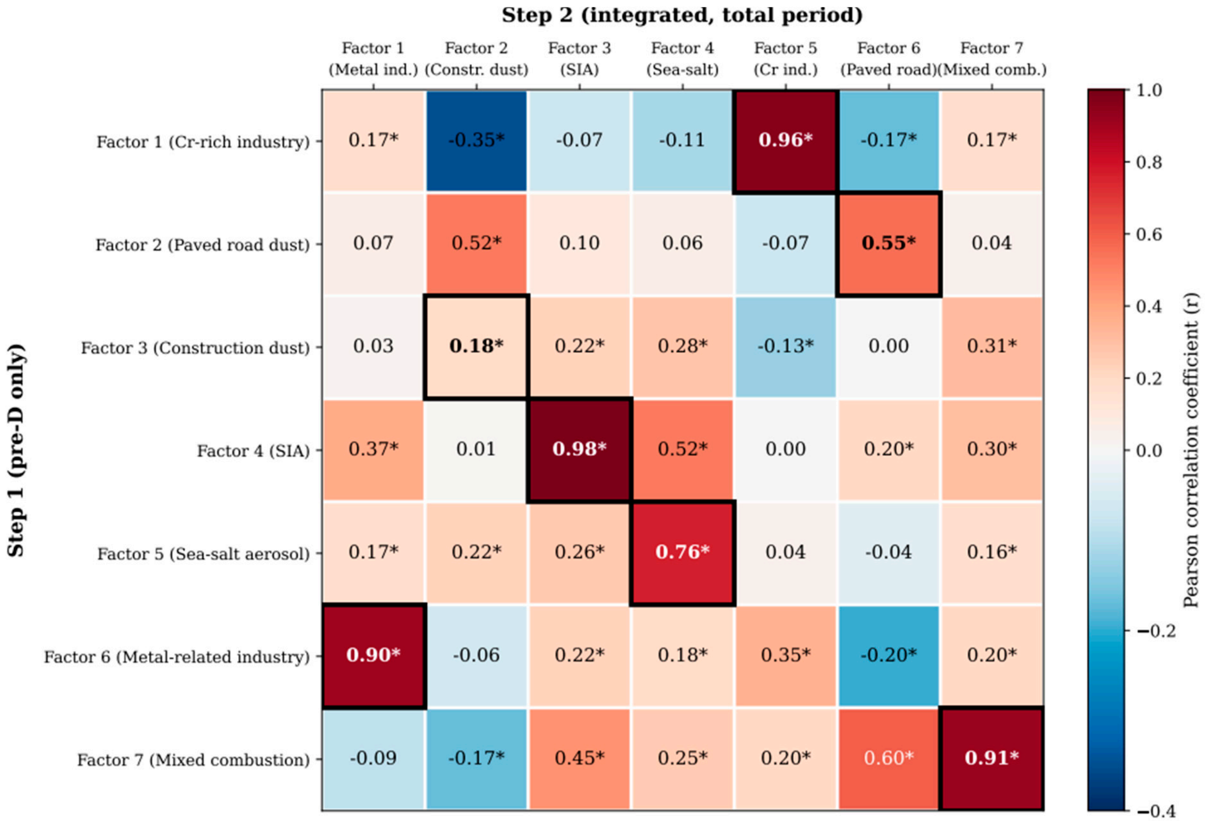

Figure S2: Heatmap for Pearson correlation coefficients between Step 1 (pre-D-only) and Step 2 (integrated) PMF factor contribution time series, overlapping period (2018–2020).

* $p < 0.05$. Matched pair determined by jointly considering the correlation coefficient, marker profile similarity, and source interpretability; for six of seven factors, this coincided with the highest r in the row, while construction dust (Step 1 Factor 3) was matched to Step 2 Factor 2 ($r = 0.18$) rather than the row-maximum Factor 7 ($r = 0.31$) based on marker profile and source interpretability (see Table S16).

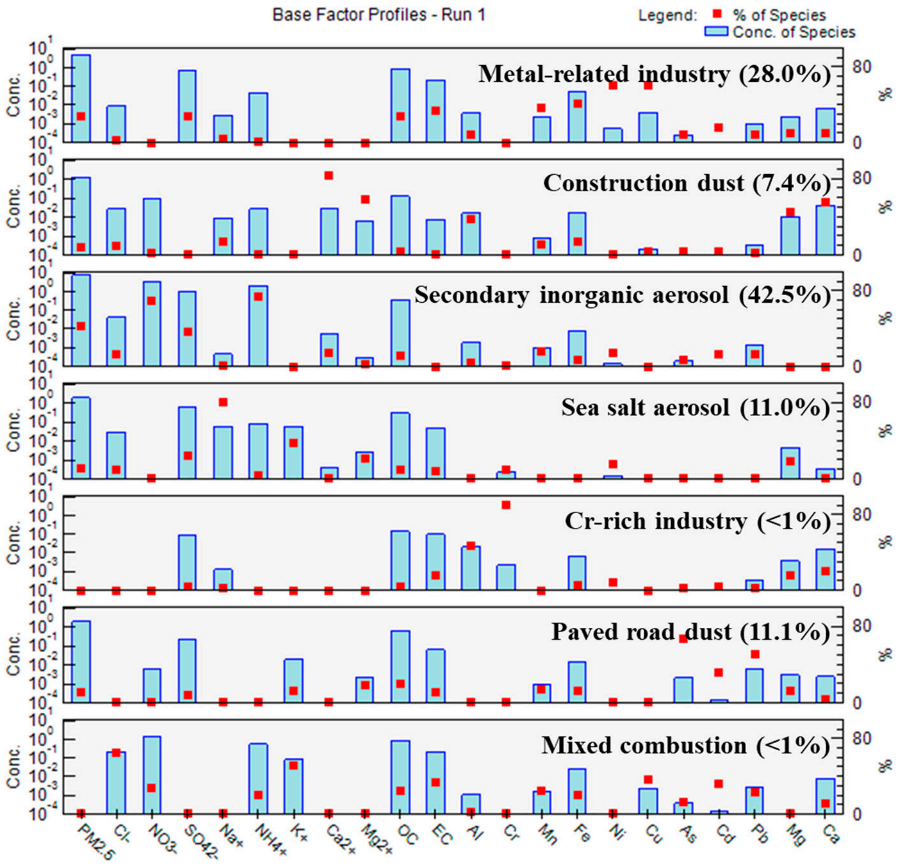

Figure S3: Source profiles and time-series contributions of the $PM_{2.5}$ factors resolved by PMF using total dataset.

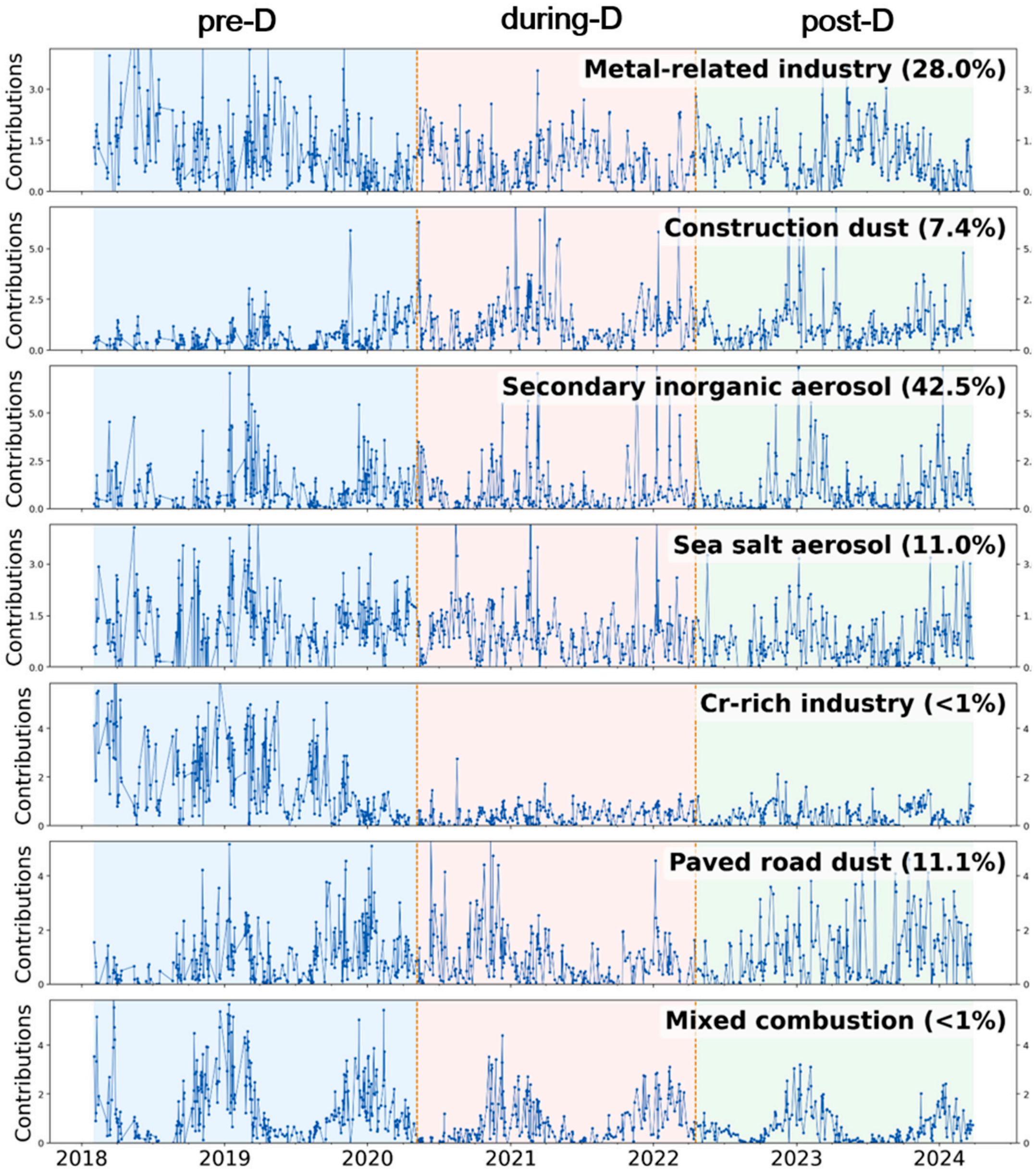

Figure S4: Temporal variations in PMF source contributions across pre-, during-, and post-D periods (2018–2024).

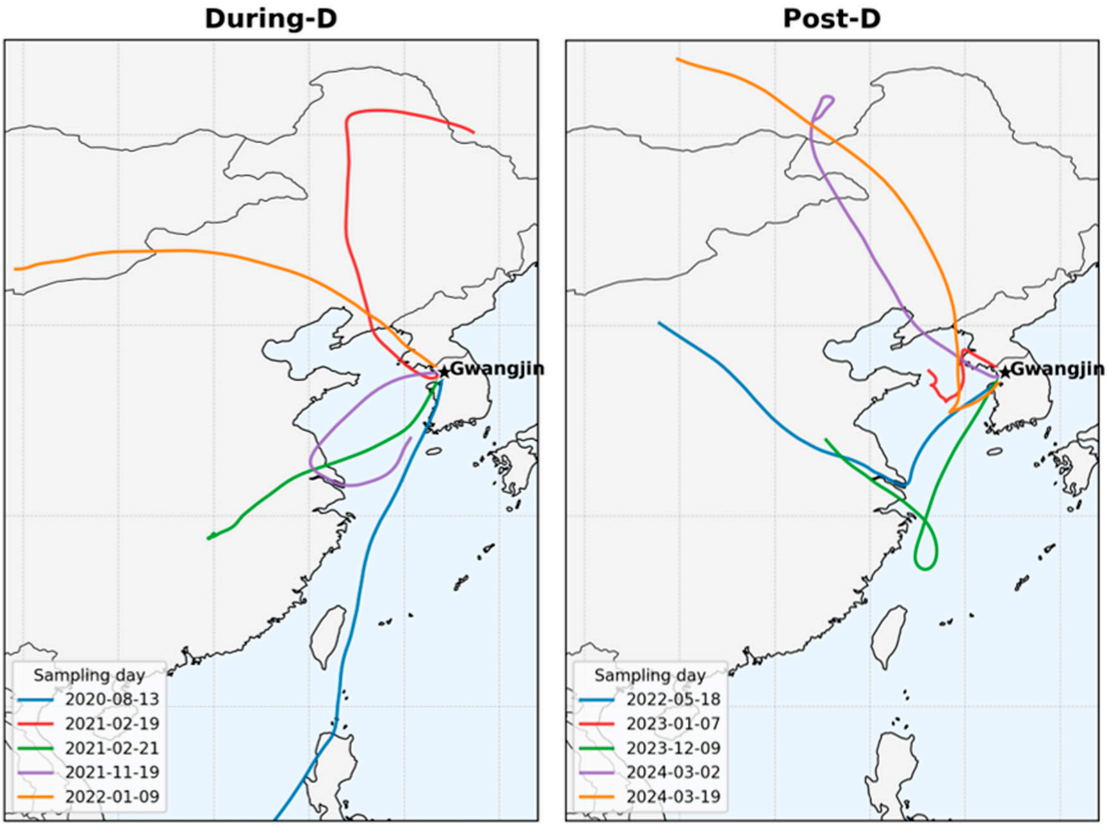

Figure S5: Seventy-two-hour backward air-mass trajectories for the five sampling days with the highest PMF-derived sea-salt factor contribution scores during the (a) during-distancing and (b) post-distancing periods.
